# Post-injury born oligodendrocytes incorporate into the glial scar and contribute to the inhibition of axon regeneration

**DOI:** 10.1242/dev.201311

**Published:** 2023-04-27

**Authors:** Jian Xing, Agnieszka Lukomska, Bruce A. Rheaume, Juhwan Kim, Muhammad S. Sajid, Ashiti Damania, Ephraim F. Trakhtenberg

**Affiliations:** Department of Neuroscience, University of Connecticut School of Medicine, 263 Farmington Avenue, Farmington, CT 06030, USA

**Keywords:** Axon regeneration, Oligodendrocytes, Optic nerve injury, Retinal ganglion cell

## Abstract

Failure of central nervous system projection neurons to spontaneously regenerate long-distance axons underlies irreversibility of white matter pathologies. A barrier to axonal regenerative research is that the axons regenerating in response to experimental treatments stall growth before reaching post-synaptic targets. Here, we test the hypothesis that the interaction of regenerating axons with live oligodendrocytes, which were absent during developmental axon growth, contributes to stalling axonal growth. To test this hypothesis, first, we used single cell RNA-seq (scRNA-seq) and immunohistology to investigate whether post-injury born oligodendrocytes incorporate into the glial scar after optic nerve injury. Then, we administered demyelination-inducing cuprizone and stimulated axon regeneration by Pten knockdown (KD) after optic nerve crush. We found that post-injury born oligodendrocyte lineage cells incorporate into the glial scar, where they are susceptible to the demyelination diet, which reduced their presence in the glial scar. We further found that the demyelination diet enhanced Pten KD-stimulated axon regeneration and that localized cuprizone injection promoted axon regeneration. We also present a resource for comparing the gene expression of scRNA-seq-profiled normal and injured optic nerve oligodendrocyte lineage cells.

## Teaser

**Summary:** Post-injury born oligodendrocytes incorporate into the glial scar and contribute to inhibiting experimentally stimulated axon regeneration within the CNS. They are also susceptible to the demyelination-inducing cuprizone diet, which promotes axon regeneration.

## INTRODUCTION

The failure of mammalian central nervous system (CNS) projection neurons to regenerate axons disrupted by white matter injury to the brain, spinal cord or optic nerve results in permanent disabilities. Like other CNS projection neurons, retinal ganglion cells (RGCs) do not regenerate axons disrupted by optic nerve crush (ONC) (Levkovitch-Verbin, 2004; Kim et al., 2018). Because molecules found to regulate regeneration of RGC axons, such as Pten and Klf7 (Liu et al., 2010; Blackmore et al., 2012), also affect spinal cord regeneration (Blackmore et al., 2012; Du et al., 2015), the mechanisms of axonal regeneration may be similar across CNS projection neurons, while the mechanisms of their pathway finding vary. A number of intracellular and extracellular factors have been discovered to affect axon regeneration (as reviewed elsewhere; Trakhtenberg and Goldberg, 2012; Goldberg and Trakhtenberg, 2012; Andereggen et al., 2015; Mar et al., 2014; Yiu and He, 2006; Benowitz et al., 2015; Chun and Cestari, 2017), but even with manipulation of potent tumorigenic factors, <1% of axons regenerated the full distance to their postsynaptic targets (de Lima et al., 2012; Lim et al., 2016; Trakhtenberg et al., 2017b). Thus, although stimulating neuronal intrinsic mechanisms of axon regeneration was sufficient for bypassing extracellular inhibitors of axon growth that are associated with the glial scar and myelin (e.g. CSPG, MAG, NogoA, OMgp, Semaphorins; Yiu and He, 2006; Silver et al., 2014), almost all regenerating axons stall growth long before reaching targets in the brain.

Here, we investigated why the regeneration of axons that respond to regenerative treatments stalls before reaching postsynaptic targets in the brain (e.g. de Lima et al., 2012; Lim et al., 2016; Trakhtenberg et al., 2014, 2017b; Belin et al., 2015; Yungher et al., 2015; Moore et al., 2009; Yin et al., 2006; Lukomska et al., 2021). We hypothesized that the interaction of the axons (which are experimentally stimulated to regenerate) with live oligodendrocytes (which were absent during developmental axon growth) contributes to stalling axonal regeneration, even after the axons have bypassed the glial scar and grown over the pre-injury myelin. Thus, the presence of newly born oligodendrocytes within the glial scar and of surviving oligodendrocytes beyond the glial scar, may stall axons from continuing to regenerate even after they grow past the glial scar. During CNS development, axon myelination is delayed until the axons have reached their postsynaptic targets in the brain. For example, oligodendrocyte appearance and myelination of the optic nerve axons in mice begins around postnatal day 7 – over 1 week after these axons have reached their targets in the brain (Foran and Peterson, 1992; Dangata et al., 1996). Myelination then prevents sprouting and stabilizes axons during the developmental pruning period (Colello and Schwab, 1994). However, optic nerve axons experimentally induced to regenerate after injury can interact with newly born oligodendrocytes in the glial scar and with surviving oligodendrocytes beyond the glial scar, and become myelinated even while they are still growing (Marin et al., 2016), which eventually could contribute to stalling axon growth even beyond the glial scar. Thus, it is possible that the interaction of the axons with live oligodendrocytes, rather than the myelin debris, is primarily what stalls experimental regeneration even after axons have bypassed the inhibitory glial scar. Indeed, conditional deletion in oligodendrocytes of the neurological diseases-associated brain-specific angiogenesis inhibitors (BAIs; which bind to the axon growth-inhibitory NogoA reticulon 4 receptor, Rtn4R), was recently shown to rescue axonal growth of co-cultured neurons (Wang et al., 2022), further supporting our hypothesis that live oligodendrocytes, and not only their debris after injury, could inhibit axon regeneration.

During development and in co-culture experiments, newly formed oligodendrocytes (NFOs) have the capacity to grow sheaths and processes that can myelinate axons; however, NFOs lose this capacity as they develop into mature oligodendrocytes (Watkins et al., 2008; Marques et al., 2016; Czopka et al., 2013). Accordingly, after a demyelinating lesion, NFOs are the primary myelin-forming and remyelinating oligodendrocytes, whereas mature surviving oligodendrocytes contribute less to post-lesion remyelination (Orthmann-Murphy et al., 2020; Neely et al., 2022; Duncan et al., 2018b). Thus, the post-injury newly born myelin-forming oligodendrocytes in the glial scar are expected to interact with the regenerating axons; as such, NFOs grow sheaths, extend processes, and attempt to myelinate the regenerating axons. However, although the surviving ONC injury mature oligodendrocytes (located beyond the injury site) have reduced capacity for growing sheaths and myelinating when they matured, some of these live surviving oligodendrocytes spontaneously re-acquire myelinating capacity (Duncan et al., 2018b) and may inhibit axon regeneration. Here, we focused on the post-injury born NFOs to investigate their presence in the glial scar and the potential to inhibit axon regeneration. To test our hypothesis, we used EdU labeling and histology to investigate whether the NFOs are present in the post-ONC glial scar, and single cell RNA-seq (scRNA-seq) to analyze the effects of ONC on optic nerve oligodendrocytes. We also used cuprizone-induced injury to oligodendrocytes (which causes demyelination in studies of multiple sclerosis) during ONC (which causes axonal optic nerve injury in studies of traumatic optic neuropathy), which acutely and irreversibly disrupts the neuronal axons while modifying the extra-axonal tissue environment (Kim et al., 2018).

## RESULTS

### Post-injury born oligodendrocytes repopulate the injury site and incorporate into the glial scar after traumatic optic nerve injury

**Fig. 1.**
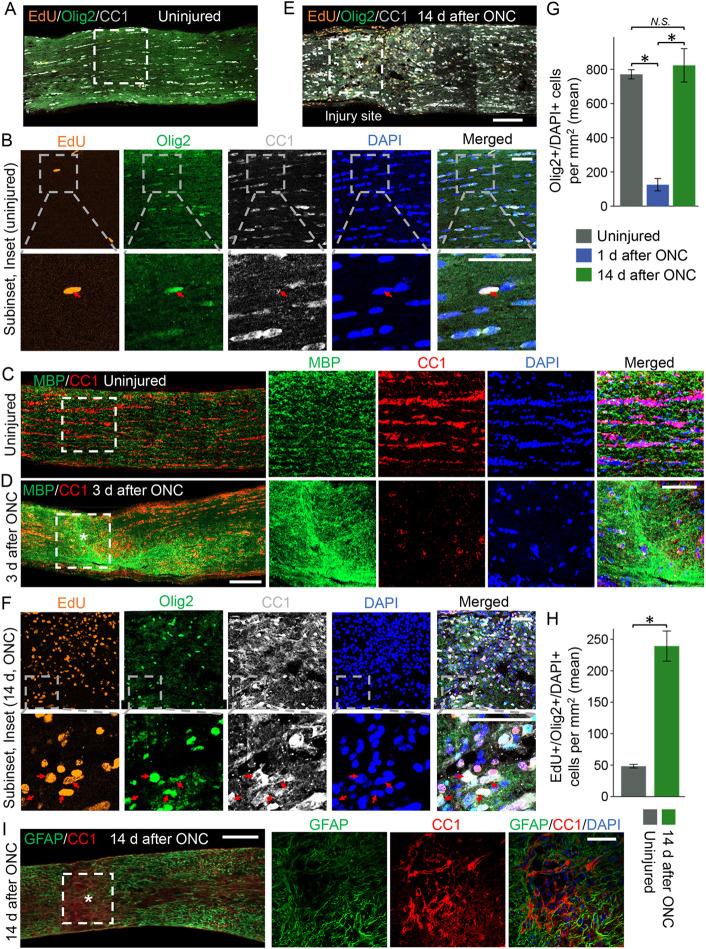
**Post-injury born oligodendrocytes repopulate the injury site in the optic nerve.** (A,B,E,F) Representative confocal images of uninjured (A,B) and injured (2 weeks after ONC, E,F) optic nerve longitudinal sections immunostained for EdU (a marker of newly born cells after EdU injections), Olig2 and CC1 (oligodendrocyte markers), and DAPI (nuclear marker), as indicated. Asterisk indicates crush site. The injury site, or equivalent region in an uninjured optic nerve, is outlined. Top row images in B and F were acquired with a 40× confocal microscope and bottom row images in B and F were acquired with a 63× confocal microscope. Red arrows indicate EdU^+^/Olig2^+^/DAPI^+^ cells. Olig2 transcription factor localizes primarily to the nucleus. CC1 labels oligodendrocyte soma, the morphology of which (i.e. extended soma and spreading processes) in the injury site is characteristic of immature and newly born oligodendrocytes (E,F), when compared with morphology in the uninjured optic nerve (A,B). Scale bars: 100 µm in A,E; 50 µm in B,F. (C,D) Representative confocal images of uninjured (C) and injured (3 days after ONC; D) optic nerve longitudinal sections immunostained for MBP (an oligodendrocyte myelin marker), CC1 and DAPI show the loss of oligodendrocytes (fewer CC1^+^ cells), and an accumulation of myelin debris from dead oligodendrocytes (MBP^+^ signal) in the injury site 3 days after ONC (D), compared with an uninjured equivalent region of the optic nerve (C). Injury site and an equivalent region in the uninjured optic nerve are outlined with dashed white lines. Scale bars: 100 µm (left image); 50 µm (right-hand images). (G) Quantification of Olig2^+^/DAPI^+^ cells in uninjured (A,B) optic nerves and injured optic nerves 1 day after ONC (Fig. S1) and 2 weeks after ONC (E,F) shows near complete loss of oligodendrocytes in the injury site 1 day after ONC and repopulation of the injury site with oligodendrocytes by 2 weeks after ONC. Data are mean±s.e.m.; *n*=3 or 4 cases for each condition (where each case is an average of three tissue sections). ANOVA with pairwise comparisons by post-hoc LSD, overall *F*=26.6, *P<0.001; N.S., not significant. (H) Quantification of EdU^+^/Olig2^+^/DAPI^+^ cells in uninjured (A,B) optic nerves and in the injury site of injured (2 weeks after ONC, E,F) optic nerves shows nearly fivefold more EdU^+^/Olig2^+^/DAPI^+^ newly born oligodendrocytes in the injury site compared with basal oligodendrocytes turnover in the uninjured optic nerve, indicating that oligodendrocytes that repopulated the injury site by 2 weeks after ONC were born after injury (as EdU injections started after ONC in injured animals and on the same day in uninjured animals, see Materials and Methods). Data are mean±s.e.m.; *n*=3 or 4 cases for each condition [where each case is an average of three tissue sections; *P<0.001 (two-tailed *t*-test)]. (I) Representative images of injured (2 weeks after ONC) optic nerve longitudinal sections immunostained for glial fibrillary acidic protein (GFAP; labels astrocytes) and CC1. The injury site is outlined with dashed white lines. Asterisk indicates crush site. Images on the right are representative confocal images of the injury site showing cellular segregation between GFAP and CC1 signals; counterstained with DAPI to label nuclei. Scale bars: 100 µm (left image); 50 µm (right-hand images).

ONC acutely and irreversibly disrupts the RGC axons in the optic nerve while modifying the extra-axonal tissue environment (Kim et al., 2018). Here, we show substantial TUNEL (cell death marker) signal and Olig2-positive cellular debris, primarily from dead oligodendrocytes, in the injury site 1 day after ONC. By comparison, TUNEL signal is absent in the optic nerve region distal to the injury site (Fig. S1). Furthermore, in the region distal to the injury site, Olig2-labeled cells, primarily oligodendrocytes (Valério-Gomes et al., 2018), are found at normal density (Fig. S1), similar to the density of Olig2-labeled cells in an uninjured optic nerve (shown in Fig. 1A,B). By 3 days after ONC, we find only a few oligodendrocytes visualized by the oligodendrocyte marker CC1 (Bin et al., 2016; Chamberlain et al., 2017; Okazaki et al., 2016; Stone et al., 2017) in the injury site (compared with uninjured control; Fig. 1C,D), whereas by 2 weeks after ONC, the injury site is repopulated with Olig2^+^/CC1^+^ co-labeled oligodendrocyte lineage cells to pre-injury density (Fig. 1E-G). Olig2 is a transcription factor that, by immunostaining, is found primarily in the nucleus (Valério-Gomes et al., 2018); however, immunostaining for CC1 enables visualization of oligodendrocyte soma, which we found in the injury site to be characteristic of immature and newly born oligodendrocytes (i.e. extended soma and spreading processes) when compared with CC1-visualized oligodendrocyte soma morphology in the uninjured optic nerve (Fig. 1A-C,E,F,I). We also administered EdU to uninjured and injured animals (starting on the 3rd day after injury and daily until 3 days before sacrifice). Two weeks after ONC, we found a near fivefold increase in newborn EdU^+^/Olig2^+^ cells, with most being co-labeled with the CC1 oligodendrocyte marker (a small fraction of cells that did not co-label with CC1 were likely oligodendrocyte progenitor cells), when compared with basal physiological turnover in the uninjured control (Young et al., 2013; Tripathi et al., 2017). This confirmed that oligodendrocyte lineage cells, which repopulated the injury site and incorporated into the glial scar, are mostly post-injury born (Fig. 1A,B,E-H). Consistent with these observations, a subset of EdU^+^ cells at 2-weeks after ONC were also positive for Fyn, which is a marker for NFOs (Osterhout et al., 1999; Zhang et al., 2014) (Fig. S2).

**Fig. 2.**
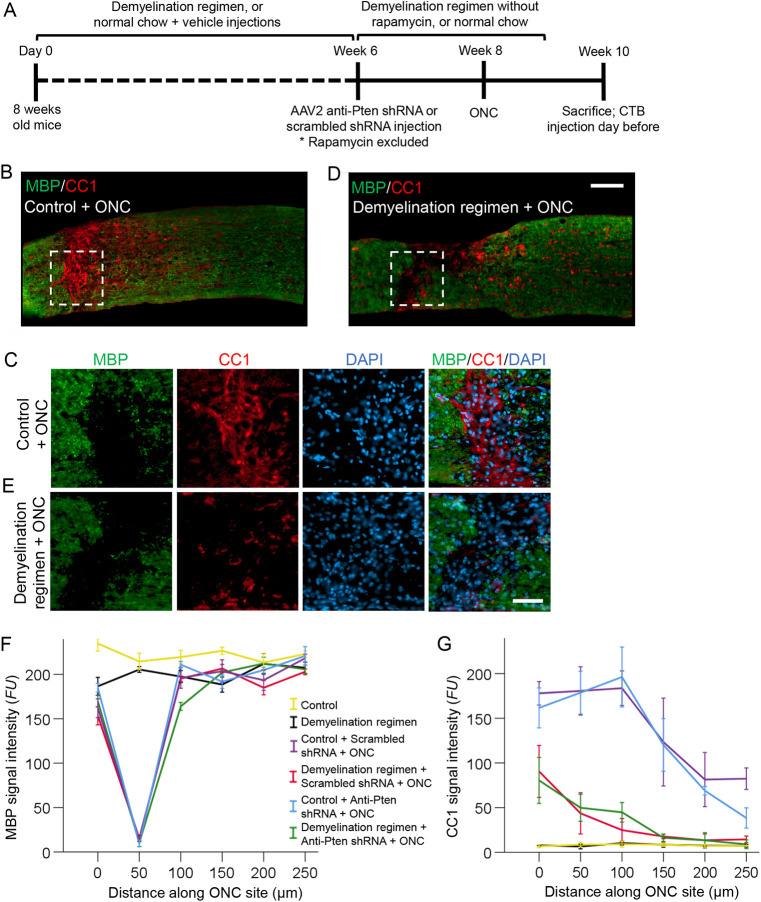
**Loss of myelin in the ONC site and the effects of the demyelination regimen on the optic nerve.** (A) Experimental timeline. Six weeks after starting the demyelination regimen, AAV2 expressing anti-Pten or scrambled control shRNA was injected intravitreally. Two weeks later, ONC was performed and mice were sacrificed 2 weeks after ONC (or 4 weeks after ONC for the data shown in Fig. 7). CTB (an axonal tracer) was injected intravitreally 1 day before sacrifice. Rapamycin was excluded from the demyelination regimen 6 weeks after starting the regimen, and the remaining regimen stopped 1 week before sacrifice. (B-E) Representative images of injured optic nerve longitudinal sections immunostained for CC1 (a mature oligodendrocyte marker), MBP (a myelin marker) and DAPI (a nuclear marker), either without (B,C) or with (D,E) the demyelination regimen. The injury site is outlined with dashed white lines. Scale bars: 100 µm in B,D; 50 µm in C,E. (F,G) Quantification of MBP (F) and CC1 (G) immunofluorescence signal intensity, represented in fluorescent units (FUs), at increasing distances along the optic nerve injury site (the region devoid of MBP and its surroundings, as shown in B-E) or along an equivalent uninjured region, across conditions as marked. Representative images for all the conditions are shown in Fig. S7 (MBP) and Fig. S8 (CC1). Data are mean±s.e.m.; *n*=4 cases for each condition. ANOVA with repeated measures, sphericity assumed, overall *F*=35.0 (MBP) and *F*=3.5 (CC1), *P*<0.001 (MBP) and *P*<0.001 (CC1). *P*-values from pairwise comparisons by post-hoc LSD are shown in Table S1A (MBP) and Table S1B (CC1).

We further characterized the injury site and expectedly found astrocytes (labeled with the glial fibrillary acidic protein, GFAP), which surrounded oligodendrocyte lineage cells (labeled by CC1, with CC1^+^ and GFAP^+^ cells segregated), within the injury site (Fig. 1I). Morphology of the CC1-labeled oligodendrocyte lineage cells within the injury site (at 2 weeks after ONC) is consistent with morphology of NFOs, including maturing small spidery oligodendrocytes (sSOs) that are primed to myelinate axons (Hines et al., 2015; Duncan et al., 2021). However, myelin basic protein (MBP) mRNA is not yet translated into protein in sSOs and is thus not detected by immunostaining (Kasuga et al., 2019) in Fig. 1F (also see control conditions after ONC in Fig. 2B-G; treatment conditions are described in later sections). These observations are consistent with oligodendrocyte progenitor cells (OPC) being present in the glial scar after injury (O'Shea et al., 2017) and differentiating into CC1^+^ NFOs (Barnabé-Heider et al., 2010), as we show that post-injury born oligodendrocyte lineage cells repopulate the injury site 2 weeks after ONC.

### Spontaneous clearance of myelin debris from the injury site by 2 weeks after ONC

**Fig. 3.**
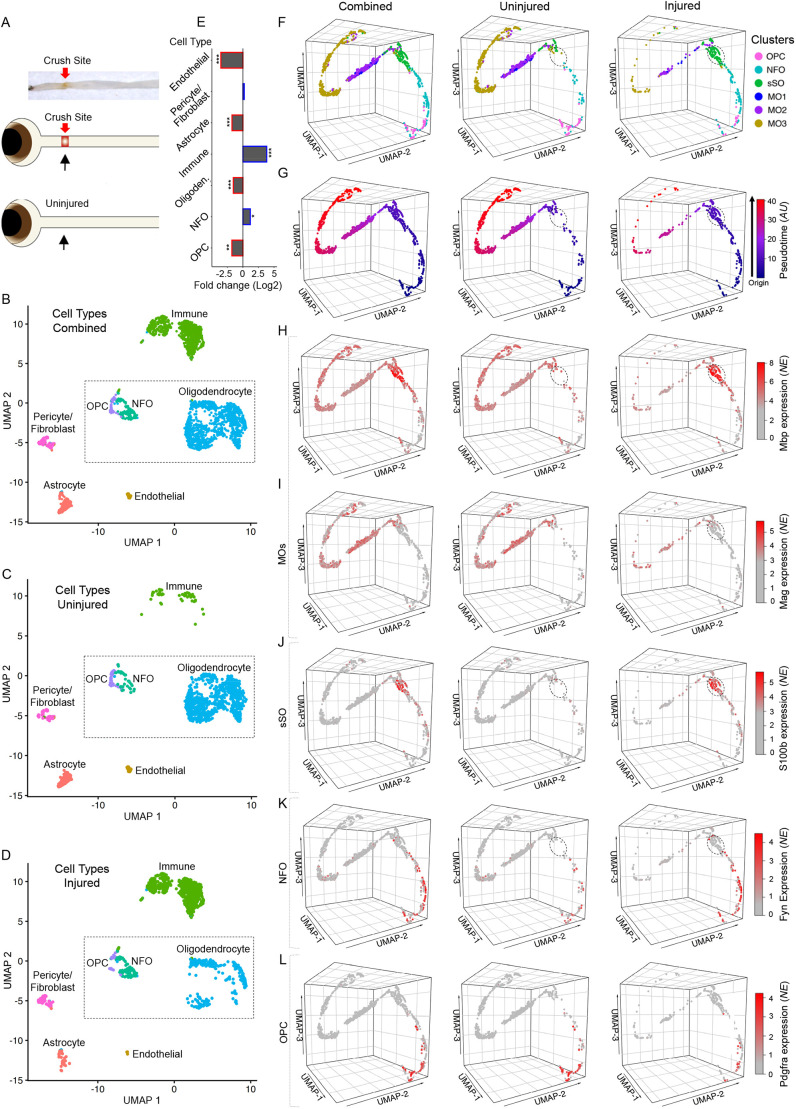
**Single cell transcriptome profiling of oligodendrocyte lineage in the uninjured and injured optic nerves.** (A) Experimental design: (Top panel) a representative image of a resected optic nerve with a red arrow indicating the crush site from where the tissue samples were dissected for scRNA-seq. (Middle and bottom panels) Cartoon representations of the injured and uninjured samples analyzed by scRNA-seq. (B-D) Two-dimensional Uniform Manifold Approximation and Projection (UMAP) of the scRNA-seq-identified cell types from the injured and uninjured optic nerve tissues: combined (in B), uninjured only (in C) and injured only (in D). The groups of cells comprising specific cell types are color coded and labeled. The UMAPs of the expression of the respective markers used to determine the cell types are shown in Fig. S4. The dashed outline indicates oligodendrocyte lineage cells for downstream analyses (in F-L). (E) Fold changes after ONC injury in the populations of cell types that make up the optic nerve tissue, as marked. Fold change significance determined by EdgeR analysis, as indicated (***P<0.001, **P<0.01 and *P<0.05; see Materials and Methods). (F) Cluster analysis (by the Seurat algorithm, see Materials and Methods) of the cell types that make up the oligodendrocyte lineage (OPCs, NFOs and oligodendrocytes; outlined by a dashed line in B-D), represented by three-dimensional (3D) UMAPs for better visualization of all cells combined, uninjured only or injured only. Clusters comprising specific cell types are color coded and annotated, as marked (OPC, oligodendrocyte progenitor cell; NFO, newly formed oligodendrocyte; sSO, small spidery oligodendrocyte; MO, mature oligodendrocyte; the cluster sSO is circled in the UMAPs of uninjured and injured cells in E-L for better visualization). (G) 3D UMAPs of cells that make up the oligodendrocyte lineage (as in F; with all cells combined, uninjured only or injured only) color coded based on pseudotimeline analysis (by the Monocle3 algorithm; AU, arbitrary units; see Materials and Methods), which represents the relative transcriptomic changes progressing along the trajectory of OPCs differentiating into NFOs, then developing into sSOs, and finally maturing into MOs. (H-L) 3D UMAPs of cells that make up the oligodendrocyte lineage (as in F,G; with all cells combined, uninjured only or injured only), showing the expression of gene markers for respective cell types (as marked): MBP (MO and sSO, in H), MAG (MO, in I), S100β (sSO, in J), Fyn (NFO, in K) and Pdgfra (OPC; in L). Scale indicates color-coded normalized expression (NE).

Myelin debris from dead oligodendrocytes, which inhibits axon regeneration (Yiu and He, 2006; Silver et al., 2014), is not cleared rapidly from the ONC injury site. Shortly (at 3 days) after ONC, we found an increase in the MBP signal (due to the accumulation of myelin debris from the dead oligodendrocytes) and a decrease in CC1 signal (due to the death of oligodendrocytes) in the injury site (Fig. 1C,D). Later (at 2 weeks) after ONC, we found apparent loss of myelin in the optic nerve injury site (see control conditions after ONC in Fig. 2B-G; treatment conditions are described in later sections), due to phagocytosing immune cells concentrating there to clear the debris (Andereggen et al., 2015; Sellés-Navarro et al., 2001) (Fig. S3). These immune cells [which infiltrate the injury site (Andereggen et al., 2015) and clear myelin and other debris (Sellés-Navarro et al., 2001)] do not appear to invade more distal nerve regions, where severed axons undergo slow Wallerian degeneration (Kanamori et al., 2012). Accordingly, using scRNA-seq analysis (see detailed discussion of the scRNA-seq experiment in the next section), we found a 14-fold increase in the immune cell population (cluster with cells positive for Iba1, Ohsawa et al., 2000; CD45, Hermiston et al., 2003; and C1qc, Zimmerman et al., 2019) in the injury site 2 weeks after ONC (Fig. 3A-E and Fig. S4A-C). These observations are also consistent with an apparent increase of Iba1^+^ microglia/macrophages density in the injury site (with CC1^+^ and Iba1^+^ cells segregated, as expected), compared with the density of these cells in a region distal to the injury site of the optic nerve (Fig. S3). Spontaneous clearance of myelin debris from within the injury site suggests that it is not a major constituent of the persisting optic nerve glial scar, although the remaining myelin sheath that still wraps the degenerating axons by the injury site, along with myelin debris at the boundary of the glial scar, may hinder axon regeneration (Yiu and He, 2006; Silver et al., 2014).

### Changes in oligodendrocyte lineage cell subpopulations in the glial scar revealed by scRNA-seq

These data are consistent with the scRNA-seq analysis of the injury site after ONC, which showed a threefold decrease in the already small (relative to other optic nerve cell types) population of Pdgfra^+^ OPCs (Pdgfra is a marker for OPCs) (Marques et al., 2018), along with a significant decrease in the mature oligodendrocyte (MO) population (Fig. 3A-E and Fig. S4D-G). However, in the injury site after ONC, Fyn^+^ NFOs (Fyn is a marker for NFOs) (Osterhout et al., 1999; Zhang et al., 2014) increased significantly (Fig. 3A-E and Fig. S4H) and MBP^+^/S100β^+^/MAG^−^ oligodendrocytes primed to myelinate axons had emerged (encircled in Fig. 3F-K). These post-injury emerging oligodendrocyte lineage cells are negative for the MO marker MAG, but are positive for S100β, which (in addition to astrocytes) is expressed in OPCs and maturing NFOs until they contact axons and consequently downregulate S100β as they progress towards full maturation (Deloulme et al., 2004; Harlow et al., 2014) (Fig. 3F-K). These post-injury-emerging lineage cells express MBP mRNA (encircled in Fig. 3H), consistent with the CC1^+^ maturing sSOs accumulating MBP mRNA before beginning its translation into protein, which becomes detectable by immunostaining at a later stage of oligodendrocytes maturation (Kasuga et al., 2019). The CC1^+^ maturing sSOs were also negative for MBP protein by immunostaining of the injury site (see control conditions after ONC in Fig. 2B-G; treatment conditions are described in later sections). The post-injury-emerging sSOs bioinformatically grouped into a discrete subpopulation of developing oligodendrocytes (green cluster labeled sSO in Fig. 3F), that was absent in the uninjured optic nerve. A small fraction of the MBP^+^/S100β^−^/MAG^+^ MOs is also present in the injury site (Fig. 3F-J). Accordingly, the pseudotimeline trajectory shows the progression from OPCs into NFOs and then into the primed-to-myelinate MBP_mRNA^+^_:_protein^−^_/S100β^+^/MAG^−^ sSOs, and finally into the fully mature MBP+/S100β−/MAG+ oligodendrocytes (Fig. 3F,G).

In the uninjured optic nerve, there are significantly fewer NFOs, and the intermediate MBP_mRNA^+^_:_protein^−^_/S100β^+^/MAG^−^ sSO subpopulation of oligodendrocytes is nearly absent, compared with the injured optic nerve. Physiological turnover rate of the oligodendrocytes is low (Young et al., 2013; Tripathi et al., 2017), and the existence of different MO subtypes in the uninjured optic nerve is consistent with the segregation of MOs into subtypes in other CNS regions (Marques et al., 2016). In the injury site, however, there is only a small subpopulation of the fully mature MBP^+^/S100β^−^/MAG^+^ oligodendrocytes, which likely includes mostly the post-injury born oligodendrocytes that matured fully and, perhaps, only some of those that survived the injury (Fig. 3F-J). This observation is consistent with almost no surviving oligodendrocytes being detected in the injury site shortly after injury (1 day after ONC) (Fig. S1), whereas by 2 weeks after injury newly born oligodendrocytes lineage cells repopulate the injury site (compared with uninjured optic nerve; Fig. 1A,B,E-H). Taken together, the immunohistological and scRNA-seq data suggest a decrease in the MO population after ONC, with only a small fraction of the injured oligodendrocytes surviving, an increase in the NFOs and the emergence of post-injury born oligodendrocyte lineage cells in the glial scar by 2 weeks after ONC.

### Combining cuprizone-induced demyelination and traumatic optic nerve injury

In studies of multiple sclerosis, a cuprizone diet injures or kills oligodendrocytes, thereby causing demyelination (Matsushima and Morell, 2001; Bénardais et al., 2013). Because the optic nerve is one of the CNS regions that is least susceptible to cuprizone-induced demyelination (Kojima and Hayashi, 2018; Yang et al., 2009), we added rapamycin injections, which reduce spontaneous remyelination, resulting in an overall greater level of demyelination (Ohno et al., 2014; Sachs et al., 2014). After the demyelination regimen is stopped, oligodendrocytes remyelinate axons (Ohno et al., 2014; Sachs et al., 2014). Because rapamycin may reduce axon regeneration after injury (Park et al., 2008), we discontinued rapamycin injections 2 weeks before ONC (Nalbandian et al., 2015; Supko and Malspeis, 1994) (experimental timeline in Fig. 2A). Because remyelination becomes detectable only about 1 week after discontinuation of the cuprizone diet (Ohno et al., 2014; Sachs et al., 2014), we stopped the diet 1 week before euthanizing the animals. After ONC, RGC-specific knockdown (KD) of Pten (see Materials and Methods and Fig. S5) was used to stimulate a subset of RGCs to regrow axons beyond the glial scar and over myelin (Park et al., 2008; Kim et al., 2018; Yungher et al., 2015).

### Effect of demyelination regimen on traumatically injured optic nerve

The effect of demyelination regimen was consistent with previous reports, as it led to robust loss of myelin in the corpus callosum, as shown by a decrease of MBP levels (Kirkpatrick et al., 2001) along the tracts (Fig. S6A,C) and in oligodendrocytes, the somas of which are labeled by immunostaining for CC1 (Bin et al., 2016; Chamberlain et al., 2017; Okazaki et al., 2016; Stone et al., 2017) (Fig. S6B,D). A greater loss of myelin in the corpus callosum compared with the optic nerve after demyelination diet was previously attributed to the optic nerve being more resistant to cuprizone (Gudi et al., 2009; Yang et al., 2009; Kojima and Hayashi, 2018). We also observed only a 10% (*P*<0.01) decrease in the MBP signal after the cuprizone diet in the uninjured optic nerve, as well as in the uninjured segment of the injured optic nerve distal to the ONC site (where the disconnected by injury axonal segments are degenerating). Within the ONC injury site (where the glial scar forms by 2 weeks after ONC), however, loss of MBP signal was apparent even without a demyelination regimen (Fig. 2B-F, Fig. S7 and Table S1A), consistent with phagocytosing immune cells concentrating there to clear debris (Andereggen et al., 2015; Sellés-Navarro et al., 2001) (as discussed above, Fig. S3). It is possible that the differences in efficiency of myelin debris clearance between corpus callosum and the optic nerve may underlie their differential response to the cuprizone diet.

### Post-injury born oligodendrocyte lineage cells in the glial scar are susceptible to the demyelination regimen

We hypothesized that the demyelination regimen would diminish the interaction of the regenerating axons with post-injury born oligodendrocytes in the glial scar, as the demyelination regimen may injure and kill oligodendrocytes (Matsushima and Morell, 2001; Bénardais et al., 2013). By injuring NFOs, the demyelination regimen may also reduce and delay the formation of new myelin in the injury site (and possibly beyond) regardless of the extent to which it clears the pre-existing myelin and its debris. We found that the demyelination regimen indeed reduced the presence of the post-injury born CC1^+^/MBP_protein^−^_ oligodendrocytes (with extended soma and spreading processes characteristic of NFOs) within the glial scar, whereas the presence of CC1-labeled oligodendrocytes (with soma morphology characteristic of mature oligodendrocytes) in the distal to injury optic nerve region did not appear to change (Fig. 2B-G, Fig. S8 and Table S1B). Consistent with cuprizone being selectively toxic to oligodendrocytes (Bénardais et al., 2013), the demyelination regimen did not appear to decrease Pdgfra-labeled OPCs or CSPG labeling in the injury site (Fig. S9). Thus, post-injury born oligodendrocyte lineage cells in the glial scar are more susceptible to the demyelination regimen compared with those distal to the injury site located in the uninjured environment (and compared with mature oligodendrocytes in uninjured control optic nerves; Fig. 1A-C, Fig. 2G and Fig. S8).

**Fig. 4.**
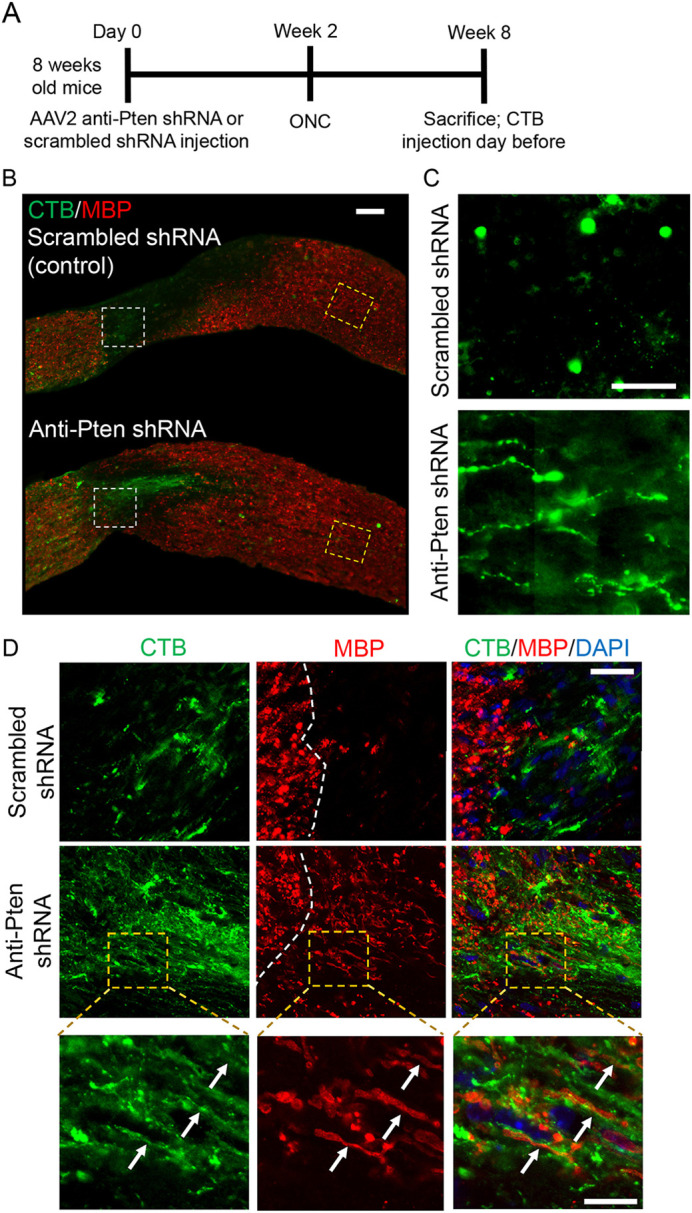
**Myelin processes in the injury site are lining the regenerating axons.** (A) Experimental timeline. AAV2 expressing anti-Pten or scrambled control shRNA was injected intravitreally. Two weeks later, ONC was performed and mice were sacrificed 6 weeks afterwards. CTB (an axonal tracer) was injected intravitreally 1 day before sacrifice. (B) Representative images of the optic nerve longitudinal sections immunostained for CTB and MBP 6 weeks after ONC, pre-treated with either AAV2 expressing scrambled shRNA (control) or anti-Pten shRNA. The injury site is outlined with a dashed white line. Scale bar: 100 µm. (C) Zoomed-in images (outlined in yellow dashed lines in B) show termination of stalled Pten KD-stimulated regenerating axons at longer distances (1.5 mm from the ONC site; lower panel), which are otherwise masked by merged MBP signal in B*.* There are no regenerating axons in control (upper panel). Exposure of the images in C was digitally increased for better visualization of the regenerating axons. Scale bar: 50 µm. (D) Representative confocal images of the areas outlined by white dashed lines in B show colocalization of MBP with the Pten KD-stimulated regenerating axons growing through the injury site (middle row)*.* There are no regenerating axons or MBP in the control injury site (top row). The dashed white line indicates the beginning of the injury site (which is on the right). Images in the bottom row show myelin processes lining the regenerating axons visualized by CTB (arrows). Scale bars: 50 µm (top and middle rows); 20 µm (bottom row).

By 2 weeks after ONC, pre-injury myelin is cleared from the injury site (even without the demyelination regimen) to the extent that MBP is barely detectable by immunostaining, while post-injury born oligodendrocyte lineage cells repopulate the injury site (Fig. 2B-F, Fig. S7 and Table S1A). Therefore, an environment such as the ONC injury site provides a unique opportunity to visualize, by immunostaining, whether the axons that are experimentally stimulated to regenerate are interacting with oligodendrocytes in the injury site as they attempt to myelinate them before the axons reach their post-synaptic targets in the brain. We have previously shown that axons that are experimentally stimulated to regenerate, stall growth before reaching their targets, even at 6 weeks after injury (Trakhtenberg et al., 2017b). Thus, to allow new myelin to accumulate sufficiently for detection by immunostaining for MBP, we analyzed the injury site 6 weeks after ONC and Pten KD-stimulated axon regeneration. Consistent with previous reports (Marin et al., 2016), confocal analysis showed the MBP signal associated with (and lining) the regenerating axons in the injury site, where MBP detection by immunostaining was otherwise bordering on noise (Fig. 4A-D).

### The demyelination regimen decreases interactions between the regenerating axons and oligodendrocyte myelin in the glial scar, and enhances axon regeneration

**Fig. 5.**
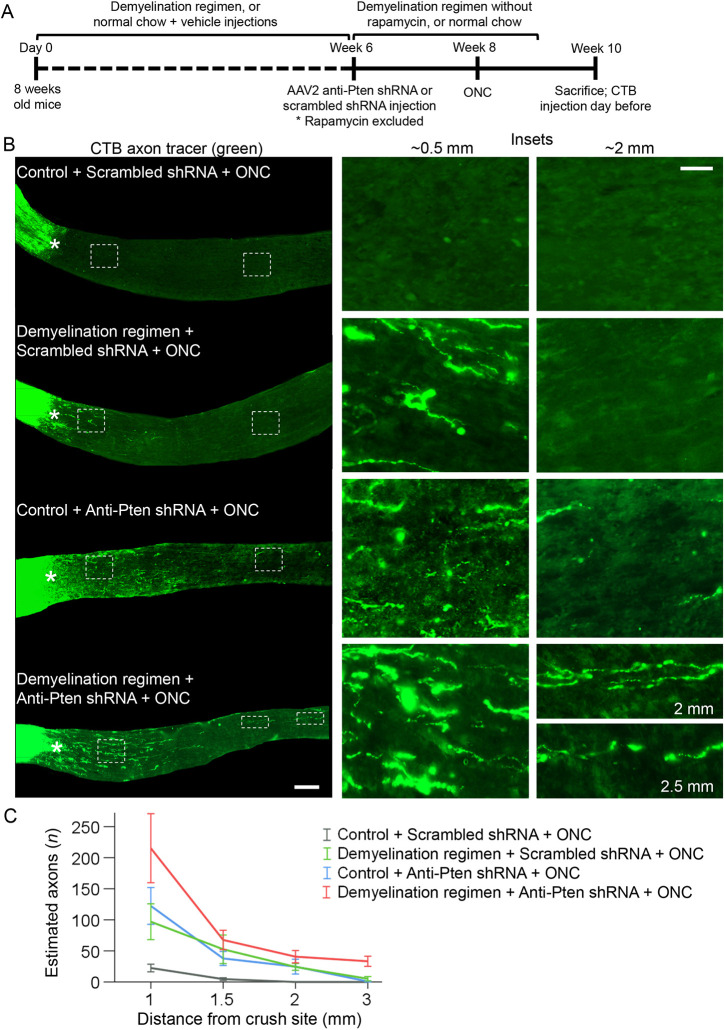
**A demyelination regimen in combination with Pten KD promotes significantly longer axon regeneration than each treatment alone.** (A) Experimental timeline. At 6 weeks after starting the demyelination regimen (or continuing on a normal chow diet in the control group) AAV2 expressing anti-Pten or scrambled control shRNAs were injected intravitreally. Two weeks later, ONC was performed and mice were sacrificed 2 weeks afterwards. CTB (axonal tracer) was injected intravitreally 1 day before sacrifice. Rapamycin was excluded from the demyelination regimen 6 weeks after starting the regimen, and the remaining regimen was stopped 1 week before sacrifice. (B) Representative images of the longitudinal optic nerve sections with CTB-labeled axons 2 weeks after ONC and pre-treatment conditions, as indicated. Asterisks indicate the crush sites. Images on the right show regions proximal and distal to the injury site that are magnified for better visualization of regenerating axons or their absence in control. Scale bars: 300 µm (left); 100 µm (right). (C) Quantification of regenerating axons visualized by CTB 2 weeks after ONC at increasing distances from the injury site across various conditions, as marked and shown in A. Data are mean±s.e.m. (*n*=5 cases for each condition). ANOVA with repeated measures, sphericity assumed, overall *F*=8.0, *P*<0.001; *P*-values of pairwise comparisons by post-hoc LSD are shown in Table S2A.

**Fig. 6.**
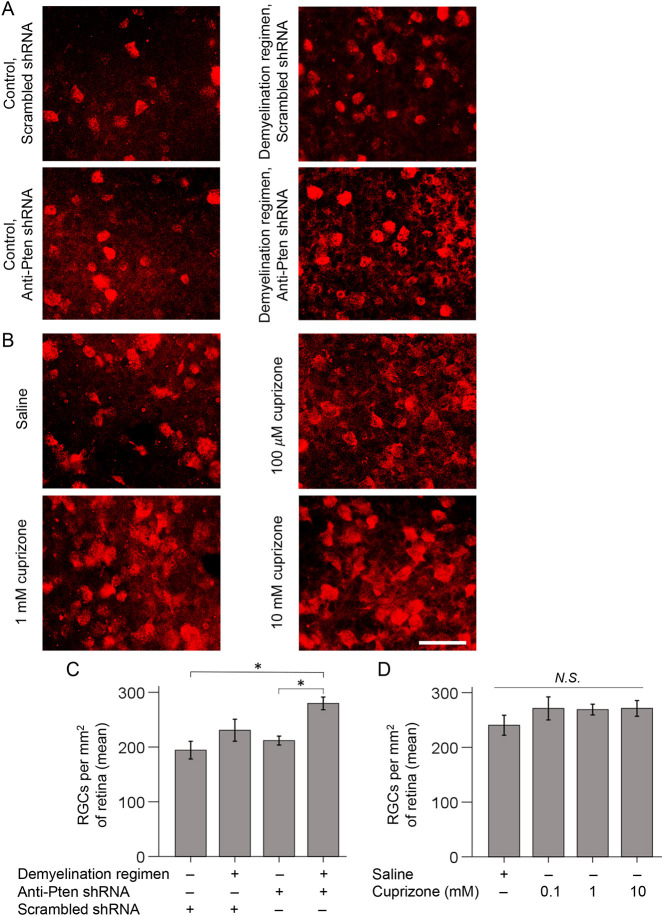
**A demyelination regimen in combination with Pten KD promotes RGC survival.** (A,B) Representative images of RBPMS-labeled RGCs in flat-mounted retinas 2 weeks after ONC and pre-treatment (A) or post-injury treatment (B) conditions, as marked (experimental timeline in Fig. 5A). Scale bar: 50 µm. (C,D) Quantification of RGC survival in retinal flat-mounts immunostained for the RGC marker RBPMS 2 weeks after ONC and pre-treatment (C) or post-injury treatment (D) conditions, as indicated. Data are mean±s.e.m. (*n*=4 or 5 cases for each condition). By ANOVA, overall *F*=3.81 (C) and *F*=0.84 (D), *P*<0.03 (C) and *P*<0.50 (D); *P*-values of pairwise comparisons by post-hoc LSD are shown in Tables S3A (C) and S3B (D). *P<0.02; N.S., not significant. Mice at the time of injury were 1 month younger for the intravitreal injection of treatments experiment (10 weeks old was typical for this assay), compared with the demyelination regimen experiment, which required prolong pre-treatment with the diet. A slightly higher RGC survival baseline in the intravitreal injection experiment compared with control used in the demyelination regimen experiment is consistent with mice being older in the latter, because older animals typically have more severe deficits after CNS injury.

**Fig. 7.**
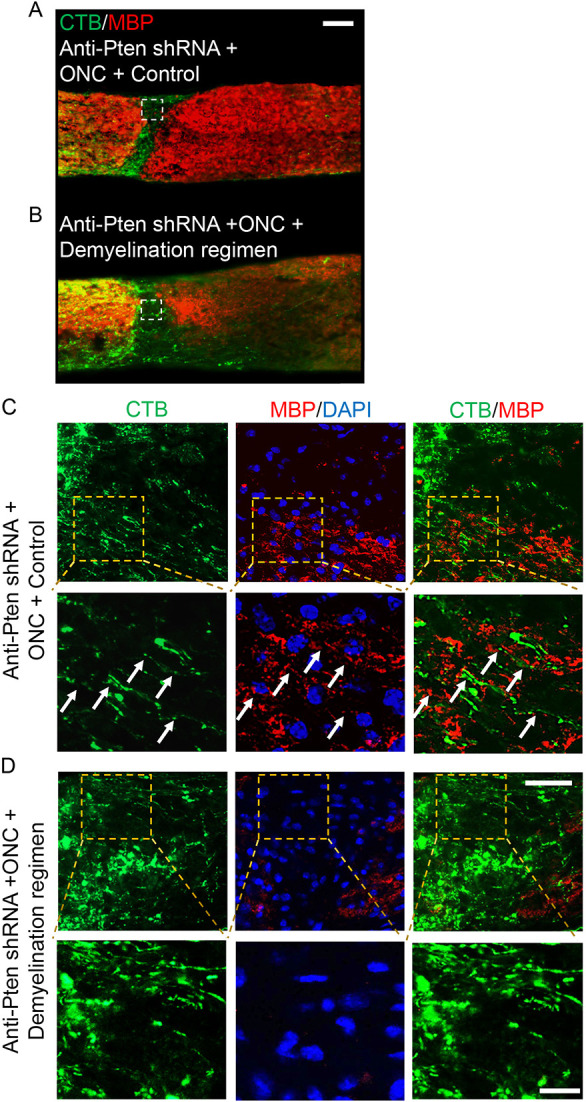
**A demyelination regimen prevents interaction of myelin processes with the regenerating axons in the injury site.** (A,B) Representative images of optic nerve longitudinal sections immunostained for MBP and CTB 4 weeks after ONC, pre-treated with AAV2 expressing anti-Pten shRNA, and either on a normal (A) or cuprizone (B) diet. The injury site is indicated by dashed white lines. Scale bar: 100 μm. (C,D) Representative confocal images of the injury site regions (outlined with white dashed lines in A,B) show Pten KD-stimulated regenerating axons growing through the injury site and interacting with myelin processes when on a normal diet (C) but not when on a cuprizone diet (D). Lower panels in C and D show the regions outlined with yellow dashed lines (upper panels) containing the regenerating axons visualized by CTB colocalizing with MBP signal (arrows in C) or the regenerating axons visualized by CTB with no adjacent MBP signal (D). Scale bars: 50 μm (top row); 20 μm (bottom row).

Next, we evaluated the effect of the demyelination regimen on axon regeneration and found significantly longer Pten KD-stimulated regenerating axons compared with Pten KD alone; even without Pten KD, some axons regenerated, albeit over a short distance (Fig. 5A-C and Fig. S2A). There was also a modest positive effect on RGC survival (Trakhtenberg et al., 2017b; Rodriguez et al., 2014) in the Pten KD/cuprizone combination compared with Pten KD alone or control (Fig. 6A,C and Table S3A). In this assay, axon regeneration is typically assessed 2 weeks after ONC, and RGCs that respond to Pten KD regenerate axons during this time-window (Yungher et al., 2015; Trakhtenberg et al., 2017b). However, this time window was insufficient for myelin on the nascent regenerated segments of the axons to accumulate to a level that is unambiguously detectable by immunostaining for MBP. By immunostaining optic nerve sections, we found that MBP was detectable on the regenerating axons without a cuprizone diet 4 weeks after ONC (Fig. 7A,C). Although the MBP signal at 4 weeks was not as robust as at 6 weeks after ONC (Fig. 4D), mice cannot be sustained on a cuprizone diet for that long because of its toxicity. Thus, as mice needed to undergo pre-treatment with the cuprizone diet in order to assess its effect on oligodendrocyte myelin interactions with the regenerating axons, we were only able to sustain mice on a cuprizone diet for up to 4 weeks after ONC. Using this approach, we found that the demyelination regimen indeed decreased oligodendrocyte myelin, and the interaction of myelin processes with the regenerated axons, in the injury site (Fig. 7).

### Localized treatment with cuprizone promotes axon regeneration

**Fig. 8.**
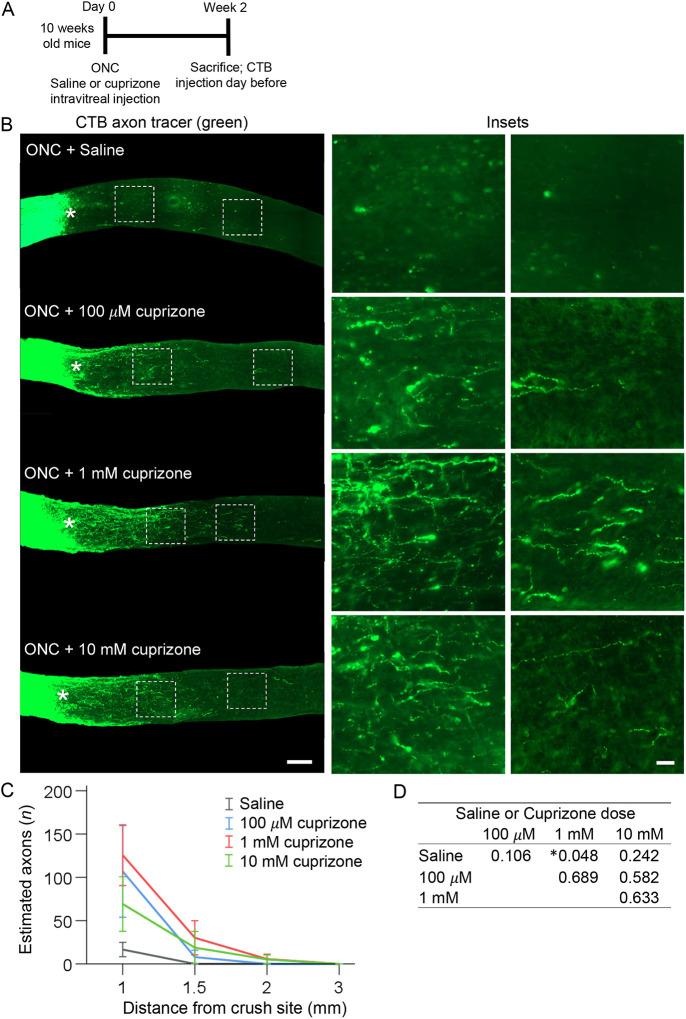
**Intravitreal injection of cuprizone after ONC promotes axon regeneration.** (A) Experimental timeline. Cuprizone or saline were injected intravitreally immediately after ONC in 10-week-old mice and 2 weeks later mice were sacrificed. CTB (an axonal tracer) was injected intravitreally 1 day before sacrifice. (B) Representative images of longitudinal optic nerve sections showing CTB-labeled axons 2 weeks after ONC, treated immediately after injury with a single intravitreal injection of vehicle or cuprizone at different concentrations, as marked. Asterisks indicate crush sites. Images on the right are of regions distal to the injury site magnified for better visualization of regenerating axons or their absence in control. Scale bars: 300 µm (left); 50 µm (right). (C,D) Quantification of regenerating axons visualized by CTB 2 weeks after ONC at increasing distances from the injury site across various conditions, as marked and shown in A. Data are mean±s.e.m. (*n*=5 cases per condition) (C). ANOVA with repeated measures, sphericity assumed, overall *F*=2.9, *P<0.03; *P*-values of pairwise comparisons by post-hoc LSD (D) are shown in detail in Table S2B.

We then asked whether a localized treatment with cuprizone facilitates axon regeneration without rapamycin and without pre-treatment (i.e. cuprizone diet prior to injury). Although (in contrast to continuous long-term administration through cuprizone diet) localized treatment is injected only once into the eye, the advantage of this is that it enables a higher concentration of cuprizone (which remains within the eye after intravitreal injection and defuses into the adjacent ONC site though the optic nerve head) to target the oligodendrocytes in the injury site without eliciting systemic toxicity. This would not be possible to achieve through the cuprizone diet, as the animals would die rapidly if systemically exposed to such a high concentration of cuprizone. Thus, a localized intravitreal injection of cuprizone was performed immediately after ONC, with 3 µl of 100 µM, 1 mM and 10 mM solutions. Two weeks after injury, all treatment groups showed a trend towards axon regeneration, but only the 1 mM dose was significant (Fig. 8A-D and Table S2B). The trend towards a positive effect on RGC survival was not significant (Fig. 6B,D and Table S3B). Our findings are consistent with previous reports that even untreated RGCs attempt, but fail, to regenerate injured axons (Sellés-Navarro et al., 2001), as hindering the interaction of the regenerating axons with oligodendrocytes (which are mostly post-injury born) in the glial scar facilitated axonal growth. These findings are also consistent with the intrinsic axon growth capacity of RGCs declining as they mature (Moore et al., 2009), because stimulating the intrinsic mechanism of axon regeneration by Pten KD in a combination with the demyelination regimen led to more robust axon regeneration than either treatment alone.

### Cuprizone does not act on RGCs directly or stimulates intraocular inflammation

**Fig. 9.**
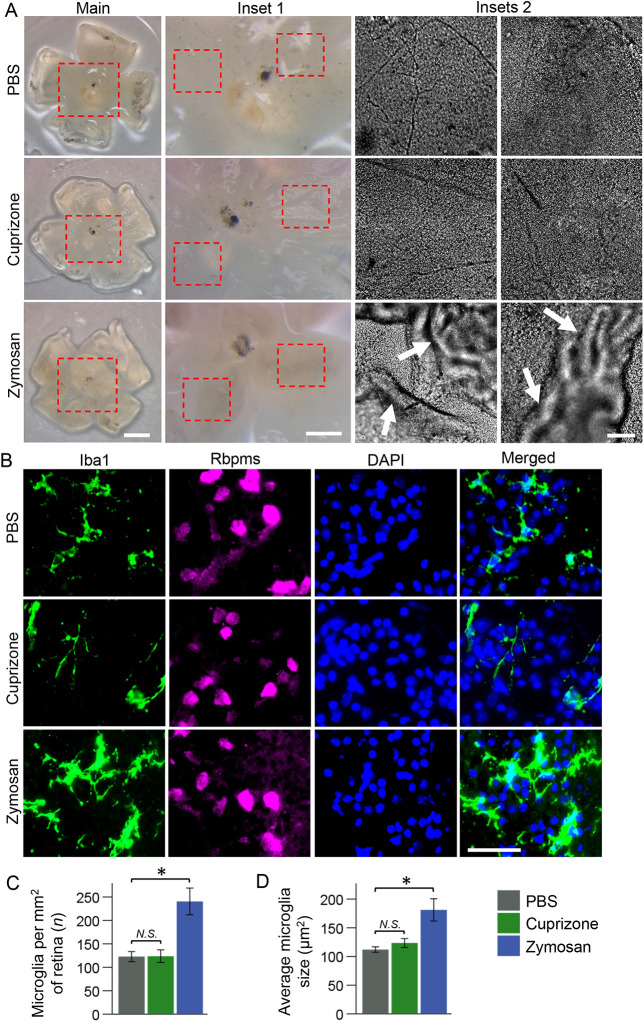
**Cuprizone does not stimulate intraocular inflammation.** (A) Representative images of flat-mount retinas from the eyes treated with PBS (control), 1 mM cuprizone and zymosan, showing examples of inflammatory fibrotic scarring (indicated by arrows) found in zymosan- but not in PBS- or cuprizone-treated eyes. Main panel and inset 1 images were acquired using a dissection microscope (Leica M205) with a HD digital camera (Leica MC170 HD). Inset 2 images were acquired using a fluorescent microscope (Zeiss, AxioObserver.Z1). Scale bars: 1 mm in main; 500 µm in inset 1; 100 µm in inset 2. (B) Representative images of flat-retinal thin sections (containing the ganglion cell layer) immunostained for Iba1 (a microglia/macrophage marker), Rbpms (a RGC marker) and DAPI (a nuclear marker) 2 weeks after ONC and intravitreal treatment with PBS (control), 1 mM cuprizone and zymosan. Images were acquired using a fluorescent microscope (Zeiss, AxioObserver.Z1). Scale bar: 50 µm. (C,D) The difference between the number of microglia per retina or microglia size (visualized by immunostaining for the Iba1 marker of microglia/macrophage) was not significantly different between PBS- and cuprizone-treated eyes, but was significant between PBS- and zymosan-treated eyes, by ANOVA with post-hoc LSD for pairwise comparisons (*P<0.01 in C and D by post-hoc LSD; overall *F*=14.4 and *P<0.01 in C; overall *F*=8.9 and *P<0.02 in D). *N*=3 samples, *n*<40 cells. Data are mean±s.e.m.

To test whether cuprizone can act on RGCs directly to affect their axon growth, we incubated adult RGCs, isolated by immunopanning for Thy1, for 5 days in culture in a defined growth medium (Trakhtenberg et al., 2014; see Materials and Methods) with varying concentrations of cuprizone. Cuprizone was not toxic to RGCs, but did not promote axon growth either (Fig. S10). To test whether cuprizone stimulates intraocular inflammation, which promotes axon regeneration (Andereggen et al., 2015), we examined the retinas for inflammatory fibrotic scarring and immunostained for Iba1 (microglia/macrophage marker) after single intravitreal injection of cuprizone (1 mM), zymosan (which promotes axon regeneration through stimulating intraocular inflammation; Andereggen et al., 2015) or PBS. We found evidence of inflammatory fibrotic scarring in zymosan-, but not PBS- or cuprizone-, treated groups (Fig. 9A). We also found significant increase in microglia/macrophage number per retina and area per cell (as activated microglia/macrophage change morphologically) in the zymosan-treated group, but no change in either parameter in the cuprizone-treated group, compared with the PBS control treated group, was found (Fig. 9B-D). These data demonstrate that cuprizone-promoted axon regeneration was not due to inflammation.

### Post-injury born oligodendrocytes express axon growth-inhibitory membrane proteins

Myelin debris-associated inhibitors of axon growth also include membrane proteins, which present themselves to the axonal stump or growth cone on the surface of live oligodendrocytes as the axons attempt to regenerate after optic nerve injury, without needing to be exposed to axonal receptors specifically on myelin debris only after the death of the oligodendrocyte. Such myelin-associated inhibitors that are presented on the surface of oligodendrocytes include NogoA (GrandPré et al., 2000), Omgp (Wang et al., 2002), and Mag (Mukhopadhyay et al., 1994). BAIs also were recently discovered to inhibit axon growth by live oligodendrocytes co-cultured with neurons (Wang et al., 2022). Furthermore, several members of the semaphorin family of axon guidance ligands, expressed on the surface of oligodendrocytes, can also inhibit axon regeneration after CNS injury (Moreau-Fauvarque et al., 2003; Ueno et al., 2020; Goldberg et al., 2004). Thus, we investigated whether these inhibitors of axon growth are expressed by post-injury born immature oligodendrocyte lineage cells.

**Fig. 10.**
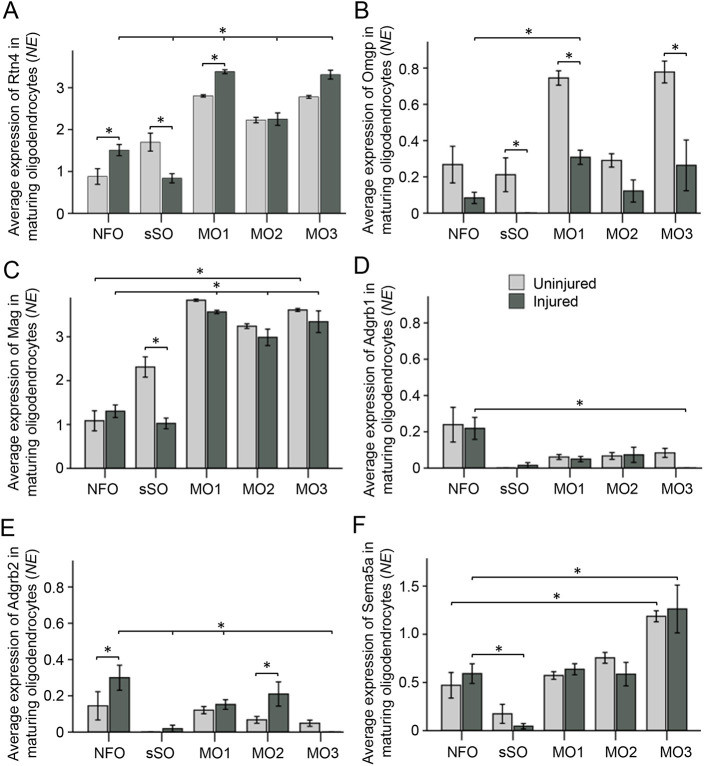
**Post-injury born oligodendrocytes express genes encoding axon growth-inhibitory membrane proteins.** Cell types are defined in Fig. 3F by cluster analysis of scRNA-seq data, including mature oligodendrocyte (MO) subtypes 1-3 (which are also found in other CNS regions; Marques et al., 2016). (A) NogoA (Rtn4) expression levels were significantly higher in the NFOs and in MO1 (but lower in the sSOs) from the injured compared with the uninjured conditions; within the injured condition, expression levels were significantly higher in the MO1-3 (but lower in the sSOs) compared with NFOs (*P<0.01). (B) Omgp was expressed at significantly higher levels in MO1 compared with NFOs within the injured condition (*P<0.03), but was expressed at significantly lower levels in the injured compared with uninjured sSOs, MO1 and MO3 (*P<0.05). (C) Mag was expressed at a significantly higher levels in MOs compared with NFOs within the uninjured and injured conditions (*P<0.01), and although its average expression is higher in the sSOs in the uninjured compared with injured condition, there were nearly threefold more sSOs in the injured compared with uninjured condition (see Fig. 3I). (D) BAI1 (Adgrb1) was expressed at significantly higher levels in the NFOs compared with sSOs, MO1 and MO2 within the injured condition (*P<0.03). (E) BAI2 (Adgrb2) expression was significantly higher in the NFOs and MO2 from the injured compared with uninjured conditions (*P<0.03), and within the injured condition expression was significantly higher in the NFOs compared with sSOs, MO1 and MO3 (*P<0.03). (F) Sema5A expression was significantly higher in the NFOs compared with sSOs within the injured condition (*P<0.01), and significantly higher in MO3 compared with NFOs within both the uninjured and injured conditions (*P<0.03). Significant differences (*P-*values) were determined by two-way ANOVA with post-hoc LSD pairwise comparisons. Data are mean±s.e.m. NE, normalized expression.

We found that NogoA (Rtn4) was expressed in all stages of oligodendrocytes maturation (see Fig. 3F), including in post-injury born NFOs. Rtn4 expression levels were significantly higher in the NFOs and in the MOs (but not in the sSOs) in the injured compared with uninjured conditions; within the injured condition, Rtn4 expression levels were significantly higher in the MOs (but not in the sSOs) compared with NFOs (Fig. 10A). Omgp (Omg) was expressed, but at a lesser level in the injured compared with uninjured conditions in all stages of oligodendrocytes maturation; within the injured condition, Omgp expression levels were higher in the MOs (but not in the sSOs) compared with NFOs (Fig. 10B). Mag was expressed moderately in the NFOs, but at less than half the levels in the MOs, and its expression levels were similar overall between uninjured and injured conditions, except for sSOs (in which its levels were higher in the uninjured condition; Fig. 10C). However, there were nearly threefold more sSOs in the injured compared with uninjured condition (Fig. 3I). BAI1 (Adgrb1) was expressed at significantly higher levels in the NFOs, and its expression levels were similar overall for each stage between uninjured and injured conditions (Fig. 10D). BAI2 (Adgrb2) also was expressed primarily in the NFOs, but its levels of expression were significantly higher in the NFOs and in the intermediate stage of maturing oligodendrocytes in the injured compared with uninjured conditions (Fig. 10E). BAI3 (Adgrb3) levels of expression bordered noise at all stages of oligodendrocyte maturation in both conditions. The axon growth-inhibitory Sema3A (Kaneko et al., 2006; Shirvan et al., 2002) was not expressed in the NFOs, and Sema3D was the only Sema3 family member that was expressed in the NFOs above background levels, but depending on CNS neuronal type, Sema3D may have no effect on axon growth or might have an inhibitory effect on only specific subtypes (Liu and Halloran, 2005; Liu et al., 2004; Sakai and Halloran, 2006). Axon growth-inhibitory Sema4D and Sema6D (Moreau-Fauvarque et al., 2003; Ueno et al., 2020) were expressed at low levels in the NFOs, in contrast to the several-fold higher levels in the MOs. Sema5A (Goldberg et al., 2004; Ueno et al., 2020) was the only axon growth-inhibitory semaphorin that was expressed moderately in the NFOs, although at half the levels in the MOs and two-fold higher than levels in the sSOs; its expression levels were similar overall between uninjured and injured conditions (Fig. 10F).

These data suggest that axon regeneration-inhibitory NogoA, Mag, BAI1-2 and Sema5A are moderately expressed in post-injury born immature and mature (including those that survived the injury) oligodendrocytes, whereas Omgp is expressed at a lower level in the injured condition and BAI3 expression is bordering on background levels in either condition. Thus, the axon growth-inhibitory membrane proteins NogoA, Omgp, Mag, BAI1-2 and Sema5A are expressed not only by the mature surviving oligodendrocytes, but also in live post-injury born oligodendrocyte lineage cells, which repopulate the injury site by 2 weeks after ONC and are also positioned to stall axonal regeneration.

## DISCUSSION

A major barrier in experimental axon regeneration research is that CNS neurons that respond to regenerative treatments stall axon growth before reaching post-synaptic targets (e.g. de Lima et al., 2012; Lim et al., 2016; Trakhtenberg et al., 2017b; Belin et al., 2015; Yungher et al., 2015; Trakhtenberg et al., 2014; Yin et al., 2006; Kim et al., 2018; Moore et al., 2009). Our results show that post-injury born oligodendrocyte lineage cells are present within the glial scar and participate in the inhibition of axon regeneration. We find that, ONC kills most oligodendrocytes in the injury site by 1 day after injury and their myelin debris fills the injury site (persisting at least 3 days after ONC); however, by 2 weeks after ONC, myelin debris is cleared from the injury site and it is repopulated by newly born oligodendrocytes. Thus, although myelin debris from dead oligodendrocytes is cleared, newly born oligodendrocytes incorporate into the forming glial scar, positioning them to also inhibit growth of the axons attempting to regenerate, and the live mature oligodendrocytes that survived the injury may also inhibit axon regeneration. The use of transgenic reporter mouse lines to label pre- and post-ONC oligodendrocytes in future studies could generate further insights into these observations.

NFOs have the capacity to grow sheaths and processes that can myelinate/remyelinate axons, but this capacity declines as they mature, and only some MOs that survive the demyelinating lesion contribute to remyelination (Watkins et al., 2008; Marques et al., 2016; Czopka et al., 2013; Orthmann-Murphy et al., 2020; Neely et al., 2022; Duncan et al., 2018b). We show that whereas uninjured oligodendrocytes in the optic nerve are mostly resilient to cuprizone (consistent with previously published literature; Yang et al., 2009; Kojima and Hayashi, 2018), post-injury born oligodendrocyte lineage cells in the injury site are susceptible to cuprizone, which thus reduces repopulation of the injury site by new oligodendrocytes and improves axon regeneration. Although cuprizone does not kill the surviving MOs located beyond the injury site in the optic nerve, it injures and affects them, as cuprizone elicits toxicity within the visual system (Marenna et al., 2022; Namekata et al., 2014); we found a 10% decrease in the MBP signal after the cuprizone diet in the region of the optic nerve distal to the ONC site. Thus, although the surviving ONC injury MOs (located beyond the injury site) have reduced capacity for growing sheaths and myelinating, some of them spontaneously re-acquire myelinating capacity (Duncan et al., 2018b). In addition, even without dying and releasing the inhibitory myelin debris, the surviving injury MOs may inhibit axon regeneration by presenting NogoA, Mag, BAI1-2 and Sema5A on their surface while they are alive. Future studies using, for example, an inducible OPC-conditional KO mouse model that prevents differentiation of oligodendrocytes post-injury (Duncan et al., 2018a), and the methods that specifically eliminate the surviving MOs in other ways, would provide insight into the extent to which the surviving (beyond the injury site) MOs are involved in inhibiting axon regeneration relative to the NFOs.

An alternative possibility that we have addressed is whether cuprizone may act on RGCs directly to stimulate their axonal growth. As we found no increase in axon growth of adult RGCs incubated with various doses of cuprizone even after 5 days in culture, we concluded that cuprizone does not stimulate axon regeneration by acting on RGCs directly. However, even at the higher, 10 mM, concentration, cuprizone was still not toxic to RGCs in culture, consistent with its established preferential toxicity for oligodendrocytes and not neurons. Another alternative possibility that we have addressed is whether cuprizone may promote axon regeneration by stimulating inflammation (Andereggen et al., 2015). Because we found that a single intravitreal injection of cuprizone (which was sufficient to promote axon regeneration) neither activated the marker (Iba1) of intraocular inflammation nor caused fibrotic retinal scarring, in contrast to zymosan (which activated Iba1 and caused fibrotic retinal scarring), we concluded that cuprizone-promoted axon regeneration was not secondary to inflammation.

While cuprizone does not affect astrocytes directly (Bénardais et al., 2013), it may indirectly lead to their activation (Skripuletz et al., 2013). Although we did not find an increase in astrocytic CSPG by the glial scar after the cuprizone diet, co-concurrent targeting of CSPG (Moon et al., 2001) and intravitreal injection of cuprizone, along with Pten or Klf9 KD in RGCs (Trakhtenberg et al., 2017b) may lead to more robust axon regeneration than each on its own. In future experimental animal studies, after cuprizone administration is discontinued (Ohno et al., 2014; Sachs et al., 2014), new oligodendrocytes can be born and myelinate the regenerated axons that have reached their respective postsynaptic targets. Thus, our findings indicate a new direction for repairing injured CNS axonal connections that, in the future, may involve localized, more-efficient and non-toxic treatments that will temporarily suspend interaction of the regenerating axons with newly born and surviving oligodendrocytes, until the axons reach their respective post-synaptic targets in the brain.

As a part of these studies, we have also generated a transcriptomic resource for studying gene networks in the optic nerve OPC and oligodendrocyte subtypes. We present a Subtype Gene Browser web application (in the same format as we did previously for the RGC subtypes; Rheaume et al., 2018) that provides a platform for analyzing and comparing gene expression profiles in OPC and oligodendrocyte subtypes from the uninjured and injured optic nerves in the resource dataset we generated (https://health.uconn.edu/neuroregeneration-lab/subtypes-gene-browser). This tool will assist the scientific community in the investigation of the differences between OPC and oligodendrocyte subtypes under physiological and injured-pathophysiological conditions. For example, consistent with prior literature, the Subtype Gene Browser shows highly enriched (compared with the differentiating and maturate oligodendrocytes) expression of NG2 cell markers (Pdgfra, CSPG4 and CSPG5) in the OPCs in both uninjured and injured conditions. In contrast to the axon growth-inhibitory CSPGs produced by reactive astrocytes (to which cuprizone is not toxic; Bénardais et al., 2013), CSPG4 and CSPG5 expressed by the OPC/NG2 cells do not inhibit axon regeneration (Anderson et al., 2016), and NG2 cells were reported to be permissive to axon growth (Busch et al., 2010; Jones et al., 2003; Schäfer and Tegeder, 2018). However, other studies suggest that NG2 cells can be inhibitory to axon growth (Chen et al., 2002; de Castro et al., 2005; Tan et al., 2006). Thus, the roles of NG2 cells in axon regeneration need further investigation (Busch and Silver, 2007; Ohtake and Li, 2015). The Subtype Gene Browser we have developed provides a resource for generating insights into this question.

Through which mechanism might live oligodendrocytes inhibit growth of the regenerating axons? It is possible that, when the oligodendrocytes attempt to myelinate the regenerating axons, they facilitate the interaction of the nascent axons with the myelin-associated inhibitors presented on the extended sheaths and processes. Thus, as the myelin-associated inhibitors on the debris of dead oligodendrocytes are cleared from the injury site by 2 weeks after ONC, post-injury born oligodendrocyte lineage cells emerge and provide new myelin-associated inhibitors within the glial scar, while some of the surviving MOs that re-acquire myelinating capacity (Duncan et al., 2018b) provide myelin-associated inhibitors beyond the injury site. The prominent myelin-associated proteins NogoA, Omgp and Mag (GrandPré et al., 2000; Wang et al., 2002; Mukhopadhyay et al., 1994) and the guidance ligand Sema5a (Goldberg et al., 2004; Ueno et al., 2020), all of which are inhibitors of axon growth, are membrane proteins that can be presented by live oligodendrocytes, without needing to be exposed to axonal receptors specifically on myelin debris only after oligodendrocyte death. BAIs also inhibit axon growth by live immature oligodendrocytes co-cultured with neurons (Wang et al., 2022). Indeed, we found that NogoA, Omgp, Mag, Sema5A and BAI1-2, are moderately expressed in oligodendrocytes newly born after ONC, as well as in MOs that survived the injury, through which they could inhibit axon regeneration. Alternatively, the axon-wrapping process/sheath may express some ligands only during the active wrapping period, during which they signal to the axon to stop growing.

In contrast to normal developmental axon growth, where myelination in the optic nerve begins a week after the axons have reached their targets in the brain (Foran and Peterson, 1992; Dangata et al., 1996), we showed here that in the adult optic nerve, live oligodendrocytes are available to interact with the regenerating axons while they are still growing (prior to reaching post-synaptic targets). Recent studies argued for therapeutically promoting myelination of the experimentally-stimulated to regenerate axons before they reached their post-synaptic targets (Wang et al., 2020). Our study, however, suggests that interaction of the regenerating axons with oligodendrocytes should be therapeutically suspended until the axons regenerate the full length needed to reach their targets, because live oligodendrocytes contribute to inhibition of axon regeneration and the axons have the capacity to grow full-length axons without oligodendrocytes, as they did during development.

## MATERIALS AND METHODS

### Animal use, diet and surgeries

All experimental procedures were performed with the approval of the Institutional Animal Care and Use Committee and of the Institutional Biosafety Committee at the University of Connecticut Health Center and performed in accordance with the ARVO Statement for the Use of Animals in Ophthalmic and Visual Research. Mice were housed in the animal facility with a 12 h light/12 h dark cycle (lighting on from 7:00 AM to 7:00 PM) with a maximum of five mice per cage. Wild-type 129S1/SvlmJ male mice were obtained from the Jackson Laboratory. Food and water were available *ad libitum*. To induce demyelination in the CNS, starting at 8 weeks of age, 0.3% cuprizone [bis(cyclohexanone)oxaldihydrazone] (MilliporeSigma) was mixed with normal chow (containing crushed Teklad global 19% protein rodent diet; Envigo), and mice started receiving, 5 days per week, intraperitoneal injections of 10 mg/kg rapamycin (LC Laboratories) dissolved in a vehicle solution of 5% ethanol in phosphate-buffered saline (PBS; Fisher Scientific), as described previously (Ohno et al., 2014; Sachs et al., 2014). Cuprizone was decreased to 0.2% at week 4 of the diet and then to 0.1% at week 8 of the diet. Normal chow diet and vehicle intraperitoneal injections were used as a control in age-matched mice. Rapamycin and control injections were discontinued after 6 weeks, when viruses with anti-Pten or scrambled shRNAs were injected, because rapamycin inhibits axon regeneration (Park et al., 2008). Allowing a 2-week period for clearance of rapamycin before optic nerve crush (ONC) is on the stringent side, because the half-life of rapamycin after injection is under 3 days, with complete clearance of even higher doses (than we used) from the organism in only a few days (Nalbandian et al., 2015; Supko and Malspeis, 1994). To stimulate axon regeneration, 3 µl of adeno-associated virus serotype 2 (AAV2, 1×10^12^ GC/ml; VectorBuilder) expressing anti-Pten (target sequences: 5′-GCAGAAACAAAAGGAGATATCA-3′, 5′-GATGATGTTTGAAACTATTCCA-3′, 5′-GTAGAGTTCTTCCACAAACAGA-3′ and 5′-GATGAAGATCAGCATTCACAAA-3′) or scrambled control (sequences: 5′-TCGAGGGCGACTTAACCTTAGG-3′) shRNAs were injected intravitreally (avoiding injury to the lens) 2 weeks before ONC. AAV2 is typically used in this model, because AAV2 (but not other AAV serotypes) preferentially transduces RGCs when injected locally into the eye vitreous. If untreated, RGCs die progressively over time after ONC. Thus, a standard protocol is to inject AAV2 2 weeks before ONC, which allows sufficient time for transduction and target gene KD (Yungher et al., 2015; Trakhtenberg et al., 2017b) (the timing of treatments and injury are shown in the experimental timelines in respective figures). Because remyelination becomes notable only within about 1 week after discontinuation of the cuprizone diet (Ohno et al., 2014; Sachs et al., 2014), cuprizone was excluded from the diet 1 week before sacrifice at 2 weeks after ONC (week 9 of the diet), except when mice were sacrificed at 4 weeks after ONC (in which case, the cuprizone diet was started 5 weeks before ONC and was stopped 1 day before sacrifice). Intravitreal injections of zymosan (3 µl; Z4250, MilliporeSigma) or cuprizone dissolved in saline, were performed immediately after ONC surgery in 10-week-old mice: 3 µl of 100 µM, 1 mM or 10 mM cuprizone solutions were injected; vehicle was used for control injections. Cholera toxin subunit β (CTB, 1% in 2 μl PBS; 103B, List Biological) axonal tracer was injected intravitreally 1 day before sacrifice. Optic nerve surgeries and intravitreal injections were performed under general anesthesia, as described previously (Trakhtenberg et al., 2017b; Kim et al., 2018). The animals were euthanized 2, 4 or 6 weeks after ONC for histological analysis.

### Histological procedures

Standard histological procedures were used, as described previously (Trakhtenberg et al., 2017b; Kim et al., 2018). Briefly, mice were anesthetized and transcardially perfused with 0.9% saline solution followed by 4% paraformaldehyde (PFA) at 3 days, or 2, 4 or 6 weeks after ONC injury. The optic nerves, eyes and brains were resected. The eyes were post-fixed for 2 h at room temperature and the optic nerves and brains for 24 h at 4°C. The optic nerves and brains were then transferred to 30% sucrose overnight at 4°C. The retinas (for analyzing RGC survival and fibrotic scar) were resected from the eyes and kept in PBS at 4°C. The optic nerves, flattened retinas (for analyzing microglia) and brains were embedded in OCT Tissue Tek Medium (Sakura Finetek), frozen and cryostat sectioned at 14 µm longitudinally for the optic nerves, horizontality (capturing the ganglion cell layer of the whole retina) for flattened retinas and at 20 μm coronally for the brains. The optic nerve cryosections were immunostained on Superfrost Plus glass slides (VWR International), and the brain cryosections and the retinas were immunostained, free-floating in wells (24-well plate), and then transferred onto the coated glass slides after making four symmetrical slits in the retinas for flat-mounting, and then mounted for imaging. For immunostaining, the tissues were blocked with appropriate sera, incubated overnight at 4°C with primary antibodies as indicated, then washed three times, incubated with appropriate fluorescent dye-conjugated secondary antibodies (1:500; Alexa Fluor, Thermo Fisher Scientific) overnight at 4°C and washed three times again.

### Imaging and quantifications of histological tissue preparations

CC1, MBP, GFAP, Olig2, Iba1, Fyn, Pdgrfa and CSPG signals in the optic nerves and brains were visualized by immunostaining, using antibodies for CC1 (1:500; mouse IgG2b, ab16794, Abcam), MBP (1:500; rabbit, ab40390, Abcam), GFAP (1:400; rabbit, ab7260, Abcam), Olig2 (1:400; rabbit, AB9610, MilliporeSigma), Iba1 (1:600; rabbit, 019-19741, Wako), Fyn (1:400; rabbit, PA5-119637, Thermo Fisher Scientific), Pdgrfa (1:400; rat, 16-1401-82, Thermo Fisher Scientific) and CSPG (1:600; mouse, MAB5284, MilliporeSigma), and counterstained with DAPI (1:5000; Thermo Fisher Scientific) to label nuclei. All fluorescent dye-conjugated Alexa Fluor secondary antibodies used were IgG H+L (Thermo Fisher Scientific, A-42206, A-78947 and A-31571), except for mouse IgG2b (A-21242, Thermo Fisher Scientific) used for CC1. To visualize dying/dead cells at one day after ONC, Click-iT Plus TUNEL Assay Alexa Fluor 594 Kit (C10618, Thermo Fisher Scientific) was used, according to the manufacturer's instructions. To label cells born after ONC, or during same time-period in uninjured optic nerves, EdU was injected intraperitoneally at 50 mg/kg daily starting from the 3rd day to the 10th day after ONC, and was also administered to uninjured animals at the same regimen. To visualize EdU, the Click-iT Plus EdU Alexa Fluor 555 Kit (C10638, Thermo Fisher Scientific) was used, according to the manufacturer's instructions. *Z*-stacked images of the optic nerves, with five planes at 0.5 µm intervals, were acquired using a fluorescent microscope (40×/1.2 C-Apochromat W; AxioObserver.Z1, Zeiss) and merged (ZEN software, Zeiss). Although a 2 h post-fix at room temperature is common for the optic nerves and is appropriate for most antibodies, for visualizing CC1 signal (using the antibody above) in the ONC site, it is necessary to post-fix for 24 h at 4°C. For quantification of MBP and CC1 signal in the optic nerves, average fluorescence signal intensity along the optic nerve diameter at 50 μm intervals throughout 250 μm distance spanning the injury site, or in an equivalent region of an uninjured optic nerve, was measured using the ImageJ plugin Plot Profile. Averages of signals from three longitudinal tissue sections per optic nerve were quantified for each biological replicate (*n*) per condition. Individual planes from the *z*-stacks were used for representative images. MBP and CC1 signals in the corpus callosum were visualized similarly, and average fluorescence signal intensity was measured using the ImageJ Measure tool in the middle of corpus callosum (344 µm×149 µm area) in three coronal tissue sections per brain, collected between Bregma −1.58 mm∼−0.94 mm coordinates. Averages of signals from three tissue sections were quantified for each biological replicate (*n*) per condition.

To quantify regenerated axons in the optic nerve, axons were visualized 2 weeks after optic nerve injury by immunostaining with the anti-CTB antibody (1:500; rabbit, GWB-7B96E4, GenWay) and fluorescent dye-conjugated secondary antibodies (1:500; Alexa Fluor, Thermo Fisher Scientific); 2 μl of CTB (1% in PBS) was injected intravitreally 1 day before sacrifice. No spared axons were found in controls or experimental conditions (i.e. at 2 weeks after injury, no axons were found at the region of the optic nerve most distal to the injury, which regenerating axons have not yet reached at this time point; Trakhtenberg et al., 2017b; Kim et al., 2018). Regenerated axons (defined as fibers continuous for >100 µm, which are absent in controls and are discernible from background puncta and artifactual structures) were counted manually using a fluorescent microscope (40×/1.2 C-Apochromat W; AxioObserver.Z1, Zeiss) in at least three longitudinal tissue sections per optic nerve at 0.5 mm, 1 mm, 1.5 mm, 2 mm and 3 mm distances from the injury site (identified by the abrupt disruption of the densely packed axons near the optic nerve head, as marked by an asterisk in the figures), and these values were used to estimate the total number of regenerating axons for each optic nerve (biological replicate, *n*) per condition, as described previously (Yin et al., 2006; Trakhtenberg et al., 2017b; de Lima et al., 2012). For representative images, serial fields of view along the longitudinal optic nerve tissue section were imaged as above; *z*-stacks with five planes at 0.5 µm intervals were deconvoluted, merged and stitched. Processed images of three tissue sections from the same optic nerve were the superimposed and merged using Photoshop CS6 (Adobe), shown as representative images.

To visualize in the optic nerve, we analyzed TUNEL and Olig2 1 day after ONC; CC1 and MBP 3 days after ONC; co-localization of EdU, CC1 and Olig2 or Fyn 2 weeks after ONC; segregation between CC1 and GFAP or Iba1 2 weeks after ONC; expression of Pdgrfa and CSPG 2 weeks after ONC; and colocalization of MBP and CTB signals in the optic nerve 4 and 6 weeks after ONC; and *z*-stacks with 0.5 µm intervals were acquired using confocal microscopy (63× or 40×/1.3 Achrostigmat Oil; LSM 880, Zeiss). Olig2- and EdU-positive cells were quantified per mm^2^ in three cryosections from each optic nerve. Counts were averaged per case, and three or four cases for each condition were analyzed.

For quantification of RGC survival, flat-mounted retinas were immunostained with an antibody for the RGC-specific marker RBPMS (Rodriguez et al., 2014; 1:500; guinea pig, 1832-RBPMS, PhospoSolutions) and RMPBS^+^ cells were counted as described previously (Yin et al., 2006; Trakhtenberg et al., 2017b; de Lima et al., 2012) using ImageJ software. RGCs were counted in at least four images per retina, acquired at prespecified areas using a fluorescent microscope (40×/1.2 C-Apochromat W; AxioObserver.Z1, Zeiss) as above, at ∼2 mm from the optic nerve head in four directions within the ganglion cell layer per retina, then averaged to estimate overall RGC survival per mm^2^. For visualization of fibrotic scarring, flat-mounted retinas were imaged using a microscope (Leica M205) with a HD digital camera (Leica MC170 HD), and insets were imaged using a microscope (Zeiss, AxioObserver.Z1). For quantification of microglia/macrophage, thin cryosections of the flattened retinas (containing the ganglion cell layer) were immunostained for RBPMS (as above) and for the microglia/macrophage-specific marker Iba1 (1:600; rabbit, 019-19741, Wako). Images were then acquired at prespecified areas using a fluorescent microscope (40×/1.2 C-Apochromat W; AxioObserver.Z1, Zeiss) and quantified as above using ImageJ software to estimate overall average microglia/macrophage number per mm^2^. To measure microglia/macrophage size, Iba1^+^ area for individual microglia/macrophages was quantified in µm^2^, using the Analyze Particles function of ImageJ software, and averaged per retina. Pten KD in RGCs by anti-Pten shRNA was validated as previously described (Yungher et al., 2015). Briefly, flat-mounted retinas transduced with either anti-Pten shRNA AAV2 or scrambled shRNA AAV2 were immunostained 2 weeks after transduction with an antibody for Pten (1:200; rabbit, 9559, Cell Signaling Technology) and neuronal marker βIII-Tubulin (1:500; mouse IgG2a, MMS-435P, BioLegend), and counterstained with DAPI (1:5000; Thermo Fisher Scientific) to label nuclei. *Z*-stack images of the ganglion cell layer were acquired using confocal microscopy as described above and orthogonal projections through the *z*-stack were used as representative images.

### RGC culture and immunostaining

RGCs were purified from both sexes of 5- to 10-week-old adult mice retinal single cell suspension by immunopanning for Thy1 (CD90, MCA02R, Serotec) after depletion of macrophages (using anti-mouse macrophage antibody; 1:75, AIA31240, Accurate Chemical) and washing off the nonadherent cells. RGCs were plated and cultured in defined growth medium following our established protocol (Meyer-Franke et al., 1995; Trakhtenberg et al., 2014, 2017a), with the following modifications for adult tissue: digestion in papain decreased to 10 min, amount of papain increased by 25%, only one macrophage depletion plate used for 20 min, Thy1 panning dish shaken more stringently, culture plates coated with a modified growth substrate, and growth factors and sato supplement doubled in the defined growth medium. RGCs were incubated with varying concentrations (as indicated in Fig. S10) of cuprizone (MilliporeSigma) or without it. After 5 days in culture, RGCs were fixed and immunostained with neuronal marker βIII-Tubulin (1:500; mouse IgG2a, MMS-435P, BioLegend) and DAPI (1:5000; Thermo Fisher Scientific). AlexaFluor fluorophore-conjugated secondary antibody (1:500; Thermo Fisher Scientific, A-21121) was used for fluorescent microscopy (with AxioObserver.Z1, Zeiss).

### Statistical analyses

All tissue processing, quantification and data analyses were anonymized throughout the study. The animals on demyelination or normal diet were randomly selected from within each group for experimental or control AAV2 injections, and the investigators performing the surgeries and quantifications were unaware of the treatment identities. Sample sizes were based on accepted standards in the literature and our previous experiences (Trakhtenberg et al., 2017b; Kim et al., 2018). Sample size (*n*) represents the total number of biological replicates in each condition. All experiments included appropriate controls. No cases were excluded in our data analysis, although a few animals that developed a cataract in the injured eye were excluded, and their tissues were not processed, and a few animals died. The data were analyzed by one- or two-way ANOVA with or without repeated measures, as appropriate, and a post-hoc LSD for pairwise comparisons, or using an independent samples *t*-test, as indicated (SPSS). Fold change significance in Fig. 3E was determined using the EdgeR algorithm (McCarthy et al., 2012).

### Single cell RNA-seq

Single cell RNA-seq (scRNA-seq) was performed using the methods we described previously (Rheaume et al., 2018), with modifications described below. Briefly, cells were purified from uninjured (*n*=5) and injured (*n*=5) optic nerves of 10-week-old mice of both sexes. ONC injury was performed at 8 weeks, as described above. Single cell suspension was prepared using slightly modified version of the method described above for obtaining single cell retinal suspension (in order to immunopan the RGCs). Cells were resuspended in DPBS with 0.04% BSA, and immediately processed as follows. Cell count and viability were determined using trypan blue on a Countess FL II, and 6000 cells from each sample were loaded in parallel for capture onto the Chromium System using the v2 single cell reagent kit (10X Genomics). After capture and lysis, cDNA was synthesized and amplified (12 cycles) according to the manufacturer's protocol (10X Genomics). The amplified cDNA from each channel of the Chromium System was used to construct an Illumina sequencing library and sequenced on HiSeq 4000 with 150-cycle sequencing. Illumina basecall files (*.bcl) were converted to FASTQs using CellRanger v1.3, which uses bcl2fastq v2.17.1.14. FASTQ files were then aligned to mm10 mouse reference genome and transcriptome using the CellRanger v1.3 software pipeline with default parameters (as described by Zheng et al., 2017), which demultiplexes the samples and generates a gene versus cell expression matrix based on the barcodes and assigned unique molecular identifiers (UMIs) that enable determining the individual cells from which each RNA molecule originated.

For determining gene expression, normalization of the raw counts was performed using Seurat (v. 4.0.3; Stuart et al., 2019; Hao et al., 2021) function, which divides the feature counts by the number of counts per cell and then applies natural log transformation, resulting in normalized expression (*NE*) values (Stuart et al., 2019; Hao et al., 2021). Dimensionality reduction and 2D visualizations were performed using the uniform manifold approximation and projection (UMAP) implementation (McInnes and Healy, 2018; Becht et al., 2018) in Seurat v. 4.0.3 using default parameters (Stuart et al., 2019; Hao et al., 2021). 3D visualization and pseudotimeline trajectory were obtained using Monocle3 v. 0.2.3.0 software with default parameters. Clustering of cells that make up the oligodendrocyte lineage into subtypes was also preformed using Seurat function with default parameters. For clustering, principal components of gene expression across cells were determined using the top 2000 most variable genes, selected by Seurat's FindVariableGenes function using the ‘vst’ method. Sex-specific genes (e.g. male Eif2s3y and Ddx3y, and female Xist), cell cycle genes and mitochondrial genes were regressed out during the scaling of the data, but were retained in the dataset for downstream analyses after clustering. A total of 2496 cells (equal number of cells from injured and uninjured optic nerves after subsampling) that passed quality control (QC) were used. QC filters/thresholds included the following criteria per cell: a maximum threshold of 20% mitochondrial genes expressed in the transcriptome, a minimum of 200 genes and a maximum of 150,000 UMIs (to mitigate the presence of cell doublets). Gene markers used to identify cell types were as follows (see Fig. S4): CD45, Iba1 and C1qc for immune cells (Hermiston et al., 2003; Ohsawa et al., 2000; Zimmerman et al., 2019), MAG and MBP for oligodendrocytes (Zhang et al., 2014), Pdgfra for OPCs (Marques et al., 2018), Fyn for NFOs (Zhang et al., 2014), GFAP for astrocytes (Zhang et al., 2014), Col1a1 and Igfbp6 for pericytes/fibroblasts (Zhang et al., 2014), and Vwf for endothelial cells (Sporn et al., 1986).

### Design of the website and online tools

The Subtypes Gene Browser was designed in the same format as used previously for RGC subtypes (Rheaume et al., 2018) using R and ShinyApps with R-markdown language (https://www.r-project.org/). Boxplots, violin plots and bar plots were adapted from ggplot2 R software package for data visualization (Wickham, 2009). The browser offers three types of analyses for any gene in the OPC and oligodendrocyte subtypes: means and s.e.m. (Wickham, 2009), violin plot of cell density for different gene expression levels (Verzani, 2014), and box plot for comparing medians using the Tukey box-and-whisker plot with whiskers set to 1.5 times the interquartile range (IQR) (https://www.r-project.org/; Benjamini, 1988; Chambers et al., 1983).

## Supplementary Material

10.1242/develop.201311_sup1 Supplementary information
