# Supplementary material for: Post-injury born oligodendrocytes incorporate into the glial scar and contribute to the inhibition of axon regeneration
Source: Development. 2023 Apr 27;150(8):dev201311. doi: 10.1242/dev.201311 (PMC10163352; doi:10.1242/dev.201311)
Supplement: Supplementary information [file develop-150-201311-s1.pdf]

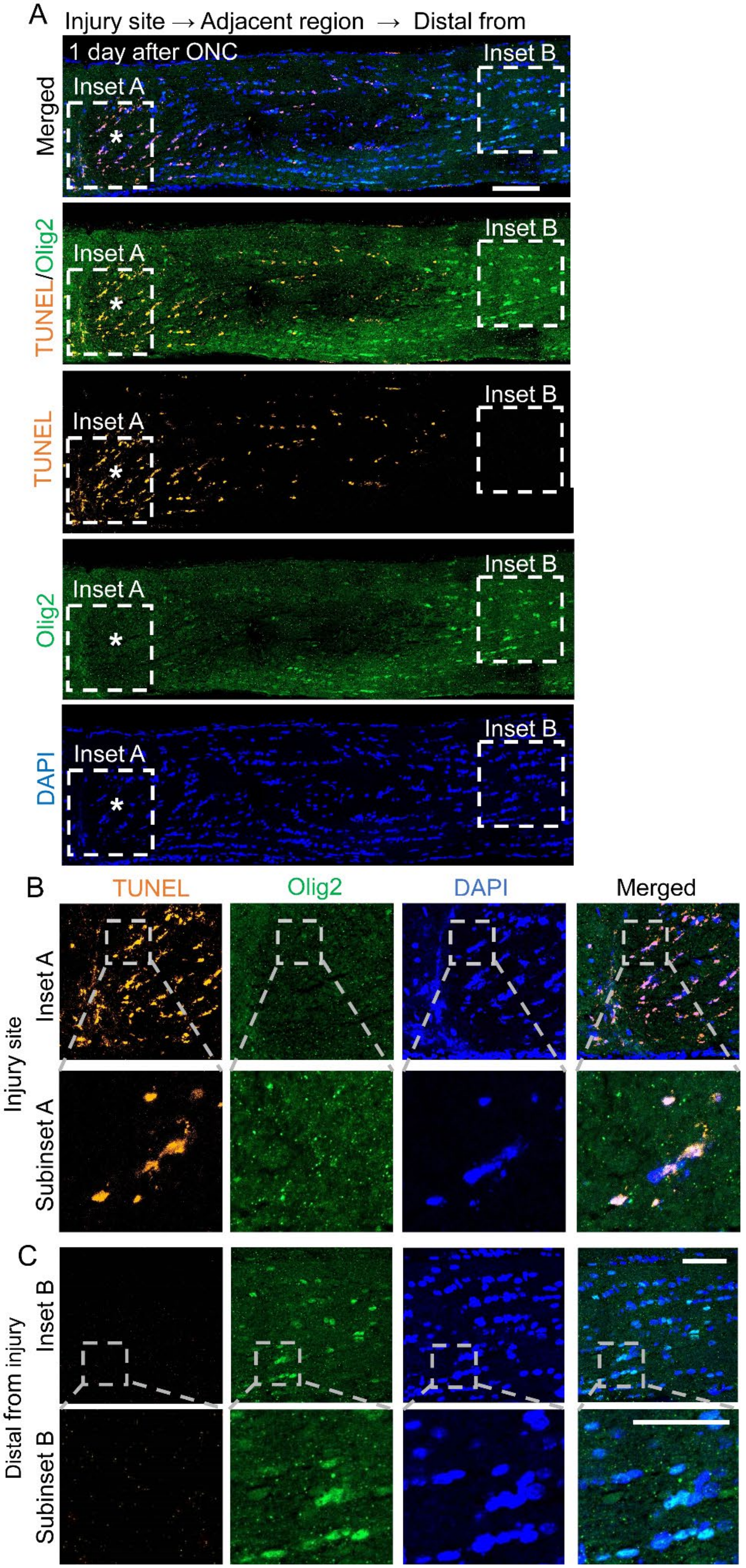
A
Injury site → Adjacent region → Distal from
1 day after ONC
Inset A
Inset B
Merged
TUNEL/Olig2
TUNEL
Olig2
DAPI
B
TUNEL
Olig2
DAPI
Merged
Inset A
Subinset A
Injury site
C
Inset B
Subinset B
Distal from injury

Fig. S1. Loss of oligodendrocytes in the optic nerve injury site at 1 day after injury. (**A**) Confocal representative images of injured (1 day after ONC) optic nerve longitudinal section immunostained for TUNEL (cell death marker, see Methods), Olig2 (oligodendrocyte marker), and DAPI (nuclear marker), as marked. (**B-C**) Injury site, and distal from injury site optic nerve region, which are outlined with dashed white lines box in *A*, are shown in insets *B* and *C*, respectively (insets acquired with 40x confocal, and subinsets acquired with 63x confocal). Normal Olig2+ cell density and no TUNEL+ cells are present distally from the injury site, whereas no Olig2+ cells and high density of TUNEL+ cells are found in the injury site. Scale bars: 100 µm main panel (*A*), 50 µm insets (*B*-*C*).

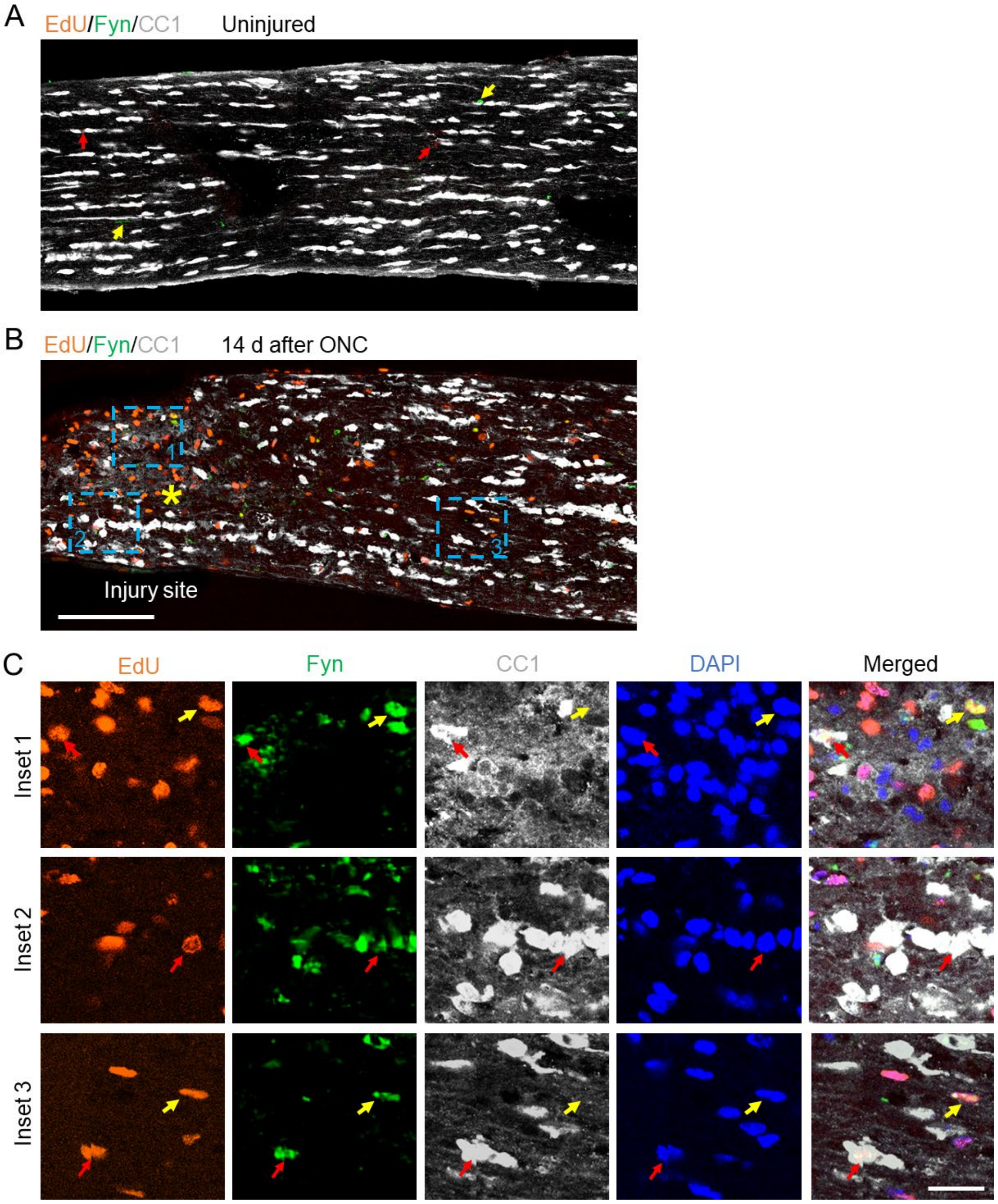

Fig. S2. Fyn+ post-injury NFOs in the optic nerve injury site. (**A-F**) Confocal representative images of uninjured (*A*) and injured (2 weeks after ONC, *B-C*) optic nerve longitudinal sections immunostained for EdU (marker of newly born cells after EdU injections), Fyn (marker of newly-born oligodendrocytes), CC1 (marker of oligodendrocytes), and DAPI

Development • Supplementary information

(nuclear marker), as marked; ★ - indicates crush site. Examples of rare instances of Fyn+ or EdU+ cells in the uninjured optic nerve (*A*) are indicated by yellow and red arrows, respectively. Representative regions of the injured optic nerve outlined with dashed blue lined boxes (in *B*) are shown as enlarged insets (*C*; acquired with 40x confocal) for better visualization. Examples of EdU+/Fyn+/CC1+/DAPI+ and EdU+/Fyn+/CC1-/DAPI+ cells (indicated by yellow and red arrows, respectively) in the injured optic nerve (*B*) are shown in the insets 1-3 (*C*). The infrequent Fyn+/CC1- cells may be immature NFOs that have not yet upregulated CC1, and/or a subset of immune cells that infiltrate the injured optic nerve, such as a subgroup of T-cells that are known to express Fyn, which also appeared as a small fraction of the scRNA-seq-analyzed cluster of the infiltering immune cells (see Supplementary Figure 4H). Scale bars: 100 μm main panels, 20 μm insets.

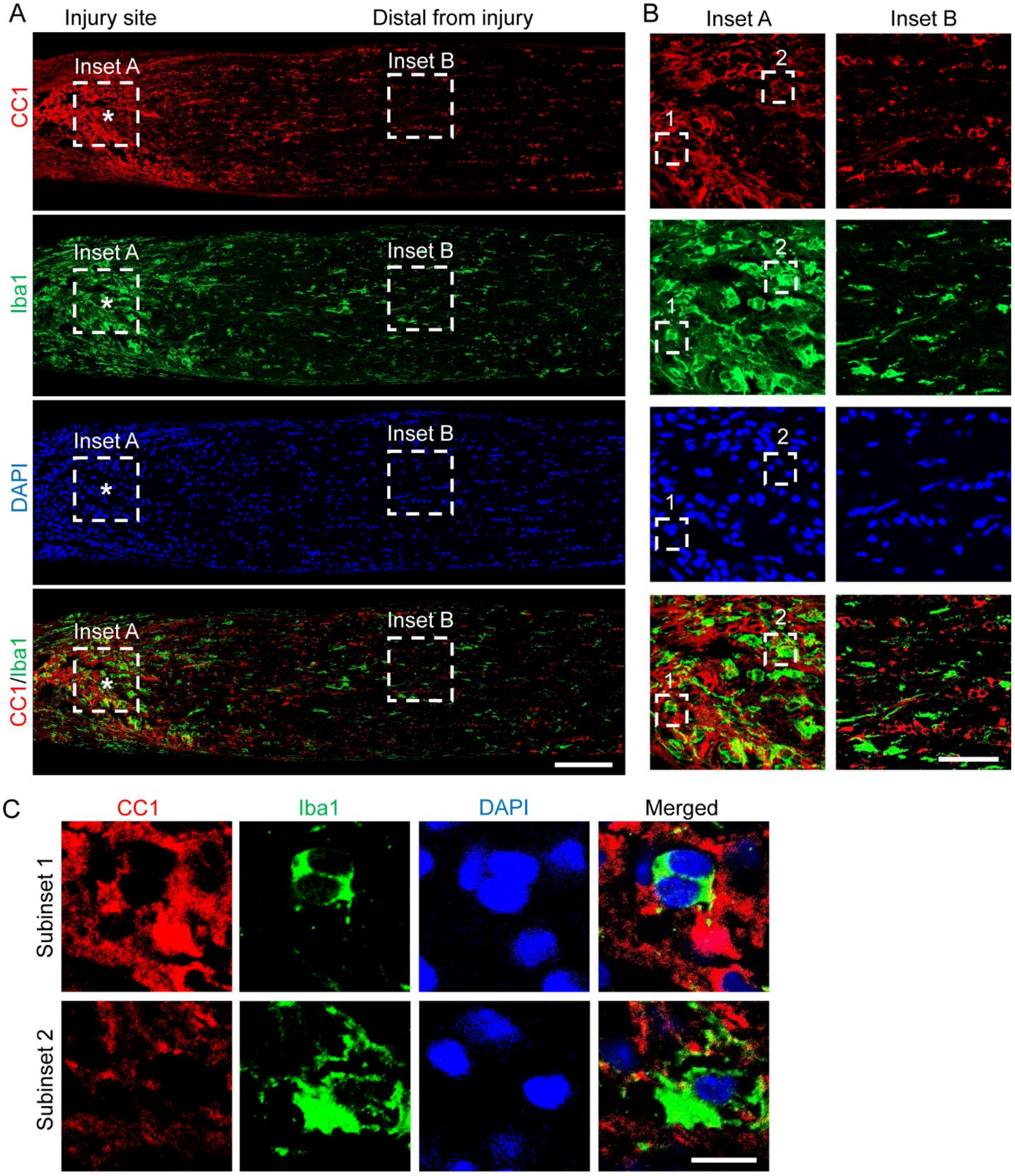

Fig. S3. Signal of oligodendrocyte marker CC1 in the optic nerve injury site does not label the microglia/macrophages. (**A**) Confocal representative images of injured (2 weeks after ONC) optic nerve longitudinal sections immunostained for Iba1 (microglia/macrophage marker), CC1 (oligodendrocyte marker), and DAPI (nuclear marker), as marked. (**B**) Insets of injury site, and distal from injury region of the optic nerve, outlined with dashed white lines box in *A*, show that both CC1-labled oligodendrocytes and Iba1-lbaled microglia/macrophages are found at increased density in the injury site, as compared to the distal from injury region of the optic nerve, but their signals do not overlap. (**C**) Subinsets, outlined with dashed white lines box in inset A (in *B*), show at higher magnification segregation between Iba1-labled and CC1-labled cells in the injury site. Scale bars: 100 μm main panels (*A*), 50 μm insets (*B*), and 10 μm subinsets (*C*).

Development • Supplementary information

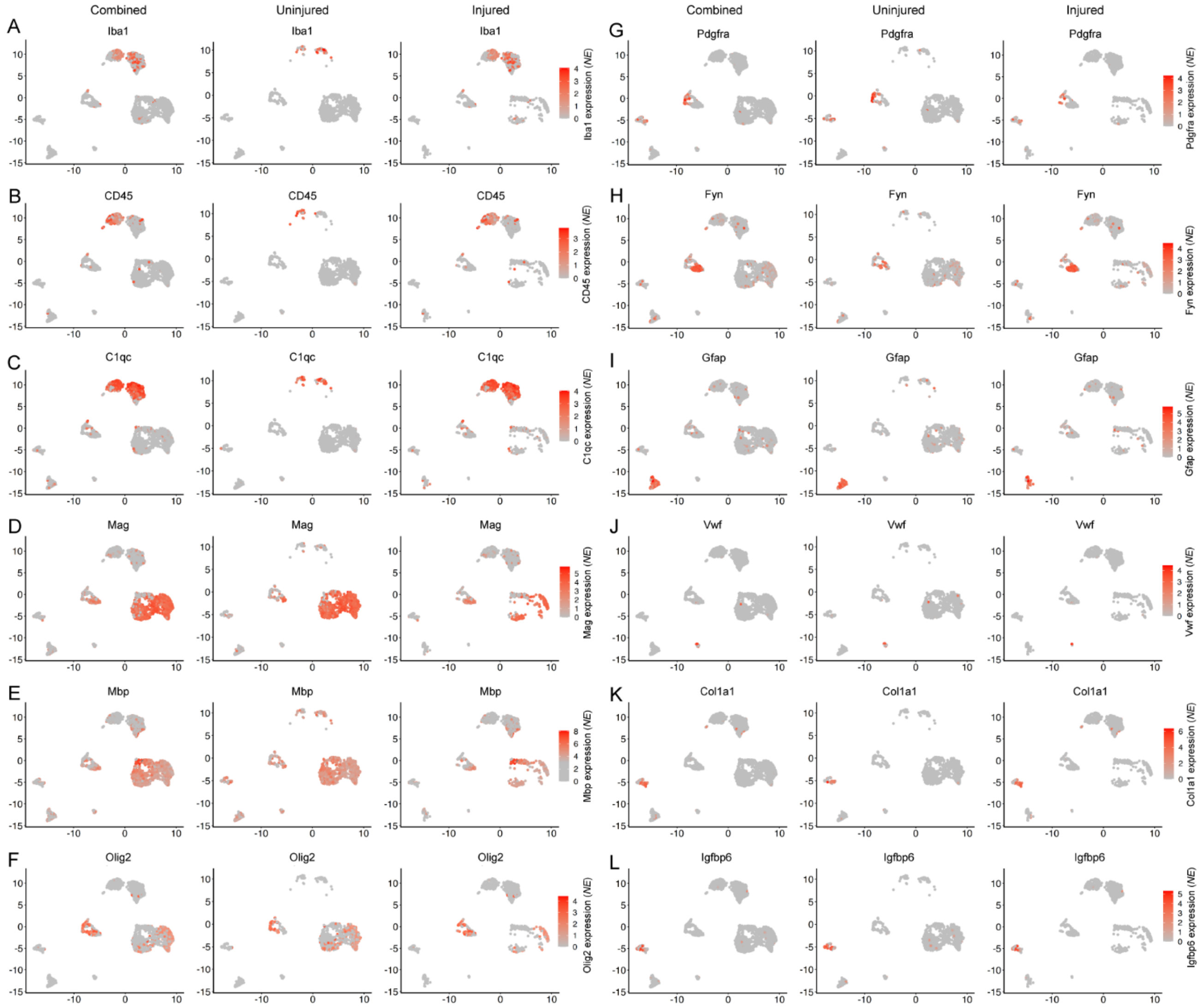

Fig. S4. Cell type markers in single cell transcriptome profiling of uninjured and injured optic nerves. (**A-L**) 2D UMAPs of the scRNA-seq-profiled cells comprising the injured and uninjured optic nerve tissues (with all with all cells combined, or uninjured only, or injured only), showing the expression of respective cell types' gene markers (as marked): Immune cell (Iba1, CD45, C1qc, in *A-C*), oligodendrocyte (MAG, MBP, in *D-F*), oligodendrocyte progenitor cell (OPC; Pdgfra, in *G*), newly formed oligodendrocyte (NFO; Fyn, in *H*), astrocyte (GFAP, in *I*), endothelial cell (Vwf, in *J*), and pericyte/fibroblast (Col1a1 and Igfbp6, in *K-L*). Scale bar, color-coded normalized expression (*NE*).

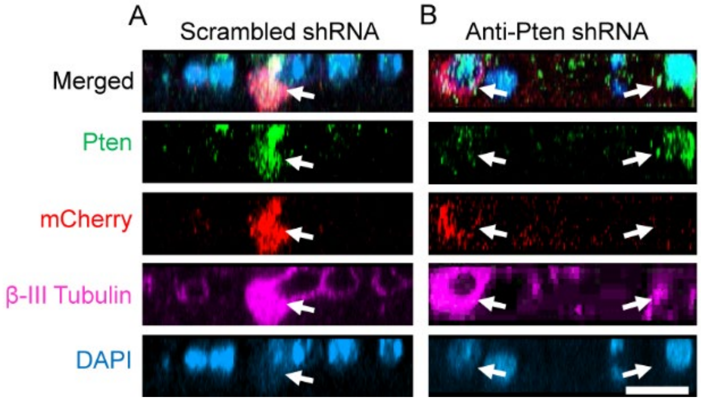

Fig. S5. Validation of Pten KD in RGCs. (**A-B**) Flat-mounted retinas transduced either with scrambled shRNA AAV2 (*A*) or anti-Pten shRNA AAV2 (*B*) co-expressing mCherry reporter, immunostained with an antibody for Pten, neuronal marker βIII-Tubulin, and counterstained with DAPI to label nuclei. Representative images show orthogonal projections through confocal *z*-stack images of the ganglion cell layer. In *A*, arrow indicates a control transduced (mCherry) RGC (βIII-Tubulin) robustly expressing Pten. In *B*, arrow on the left indicates control transduced (mCherry reporter) RGC (βIII-Tubulin) not expressing Pten, whereas the arrow on the right indicates a non-transduced (no mCherry) RGC (βIII-Tubulin) robustly expressing Pten. Scale bar, 20 μm.

Development • Supplementary information

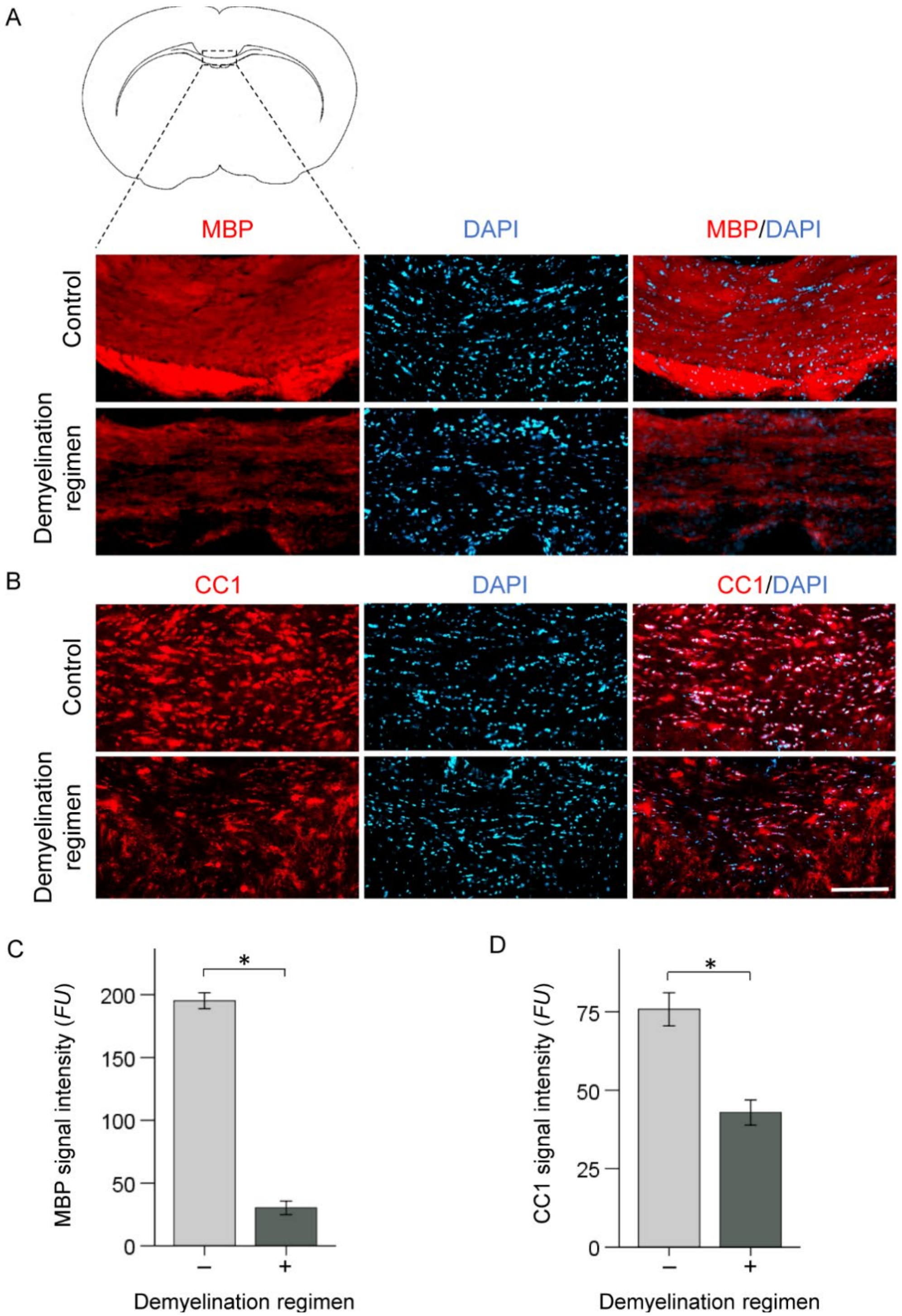

Fig. S6. Loss of myelin basic protein (MBP) and oligodendrocyte marker CC1 in the corpus callosum after demyelination regimen. (**A**) Representative coronal images of the corpus callosum immunostained for MBP without (upper panel) or with (lower panel) demyelination regimen. Scale bar, 100 μm. (**B**) Representative coronal images of the corpus callosum immunostained for oligodendrocyte marker CC1 without (upper panel) or with (lower panel) demyelination regimen. Scale bar, 100 μm. (**C-D**) Quantification of MBP (*C*) and CC1 (*D*) immunofluorescence signal intensity, represented in fluorescent units (*FU*s), in the corpus callosum, as marked (Mean ± S.E.M shown; n = 3 per group, where each case is an average of 3 tissue sections); p-values by t-test 2-tailed, * $p < 0.001$ (MBP in *C*) and * $p < 0.01$ (CC1 in *D*).

Development • Supplementary information

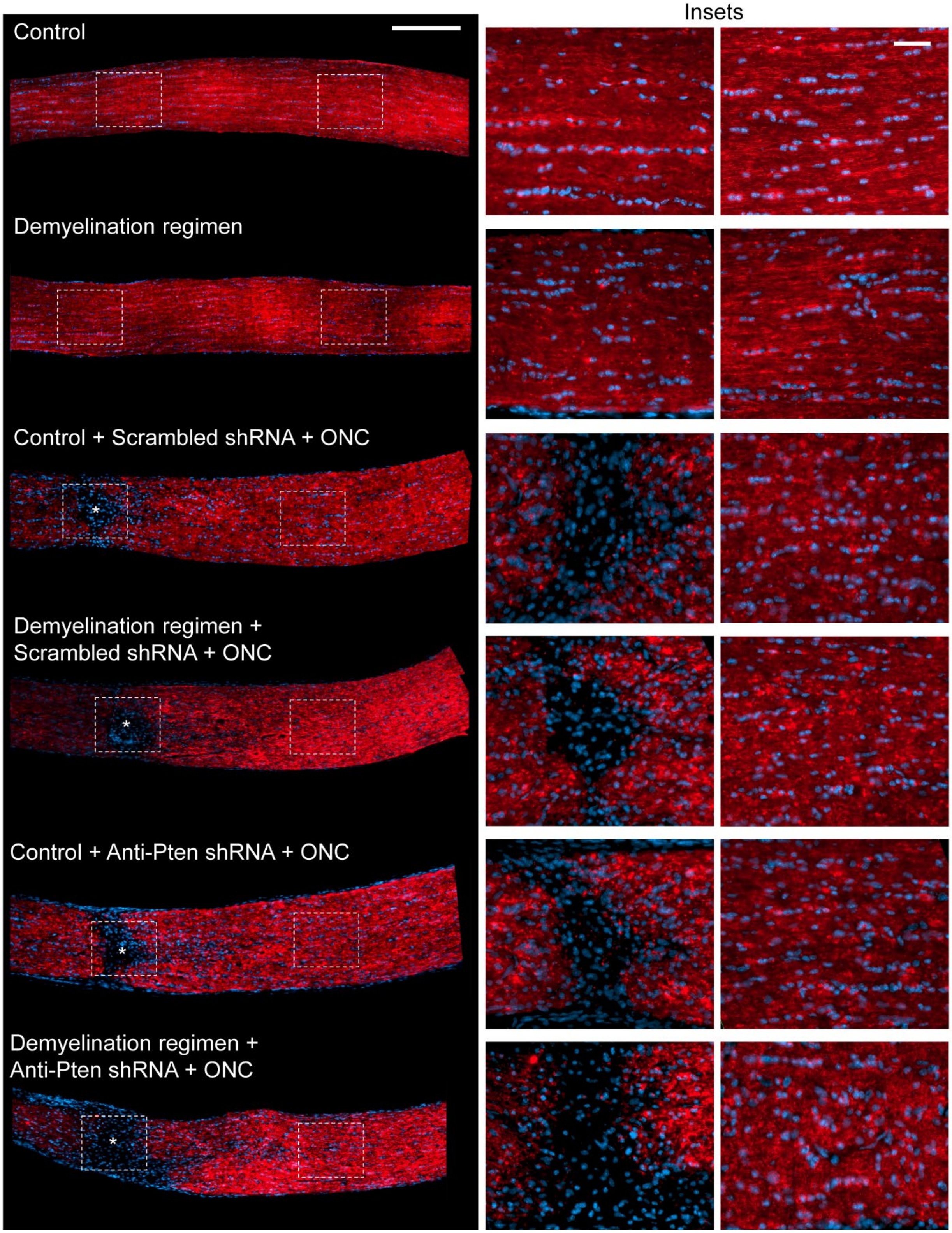

Fig. S7. Myelin in uninjured and injured optic nerves after demyelination regimen. Representative images of longitudinal optic nerve sections immunostained for MBP and DAPI 2 weeks after ONC or uninjured, with or without demyelination regimen, across various conditions as marked (experimental timeline in Fig. 2A). ⋆ - indicates crush site. Insets: Images of the uninjured or injured site, and of distal regions, are magnified for better visualization of MBP signal and DAPI-labeled nuclei. Scale bars, 200 μm (main panels), 50 μm (insets).

Development • Supplementary information

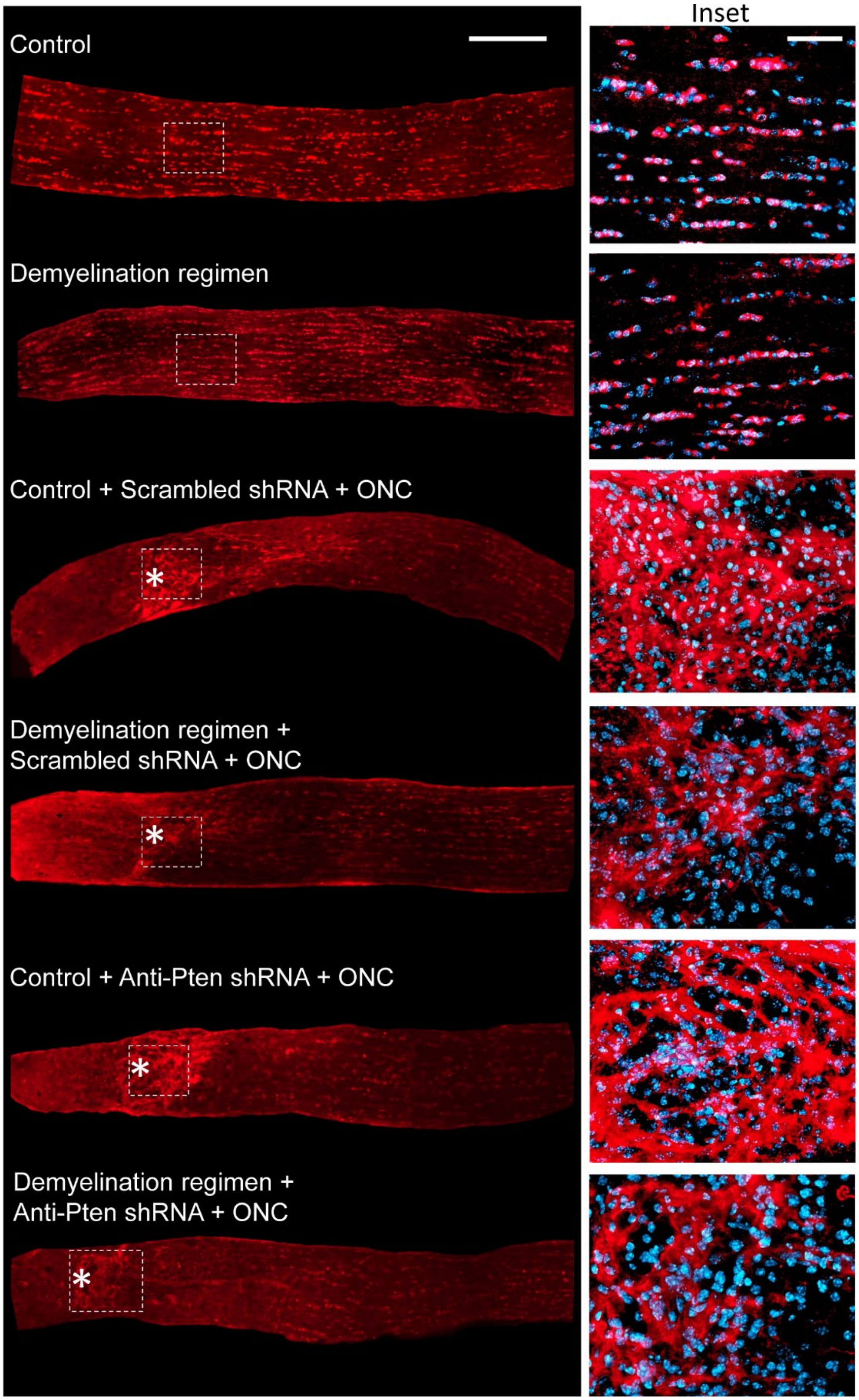

Fig. S8. Oligodendrocytes in uninjured and injured optic nerves after demyelination regimen. Representative images of the longitudinal optic nerve sections immunostained for CC1 and DAPI 2 weeks after ONC or uninjured, with or without demyelination regimen, across various conditions as marked (experimental timeline in Fig. 2A). ⋆ - indicates crush site. Insets: Images of the uninjured or injured sites are magnified for better visualization of CC1 signal and DAPI-labeled nuclei. Scale bars, 200 μm (main panels), 50 μm (insets).

Development • Supplementary information

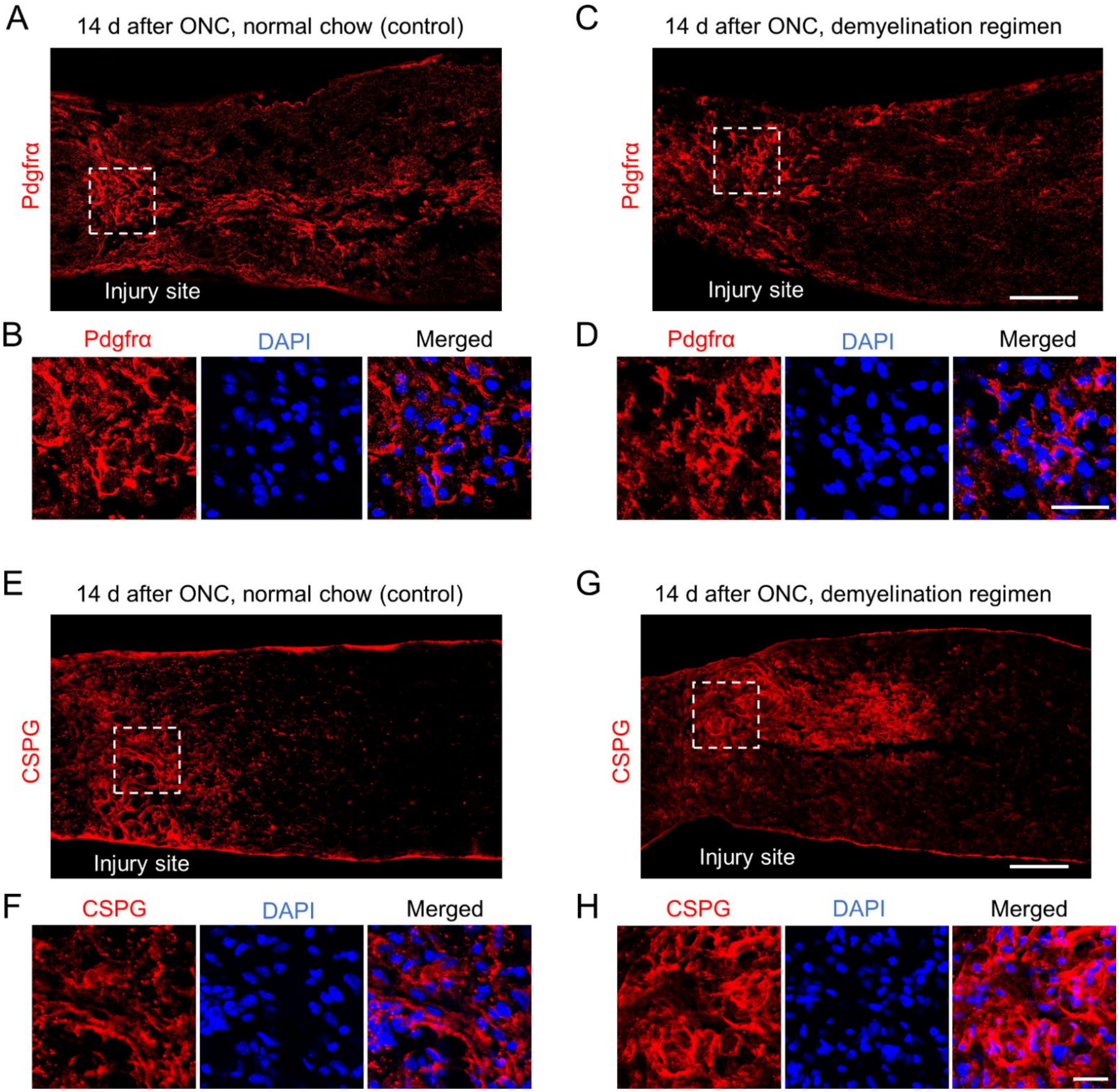

Fig. S9. Pdgfrα and CSPG in injured optic nerves after demyelination regimen. (**A-D**) Confocal representative images of injured (2 weeks after ONC) optic nerve longitudinal sections, without (*A*-*B*) or with (*C*-*D*) demyelination regimen, immunostained for Pdgfra (marker of OPCs) and DAPI (nuclear marker), as marked. Representative regions of the injured optic nerves outlined with dashed white lined boxes (in *A* and *C)* are shown as enlarged insets for better visualization of the injury site without (*B*) or with (*D*) demyelination

Development • Supplementary information

regimen (acquired with 40x confocal). There is no apparent decrease in Pdgfra-labeled OPCs in the injury site of the optic nerves with demyelination regimen, as compared to the control optic nerves with normal chow. (**E-H**) Confocal representative images of injured (2 weeks after ONC) optic nerve longitudinal sections, without (*E-F*) or with (*G-H*) demyelination regimen, immunostained for CSPG (axon growth-inhibitor expressed by reactive astrocytes) and DAPI (nuclear marker), as marked. Representative regions of the injured optic nerves outlined with dashed white lined boxes (in *E* and *G*) are shown as enlarged insets for better visualization of the injury site without (*F*) or with (*H*) demyelination regimen (acquired with 40x confocal). There was no apparent decrease in CSPG-labeling in the injury site of the optic nerves with demyelination regimen, as compared to the control optic nerves with normal chow. Scale bars: 100 μm main panels, 20 μm insets.

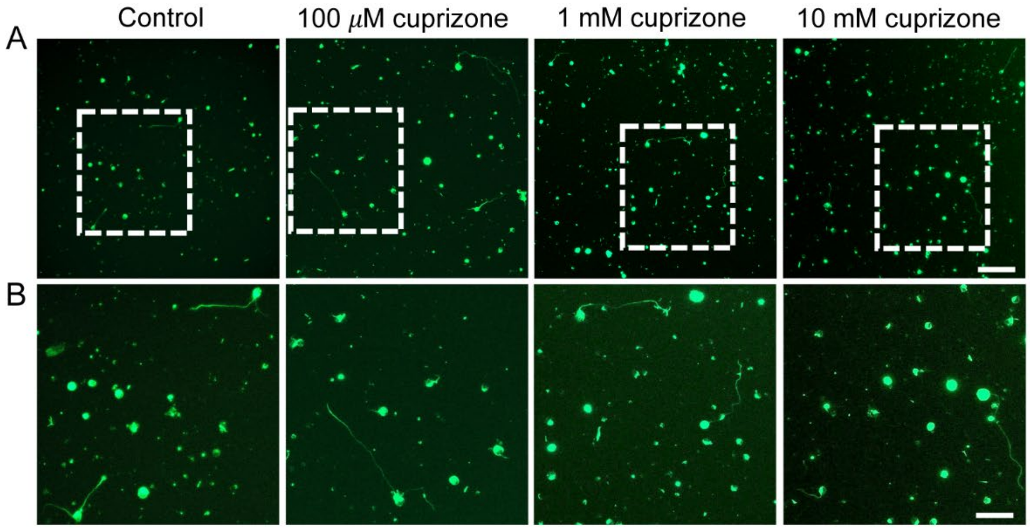

Fig. S10. RGCs incubated with varying concentrations of cuprizone. Adult RGCs, isolated by immunopanning for Thy1, immunostained with neuronal marker βIII-Tubulin after 5 days in culture in a defined growth medium (see Methods) with varying concentrations of cuprizone, as indicated. (**A-B**) There was no apparent difference in RGC density (larger field of view in *A*) or axon growth (insets in *B*) between control or cuprizone treated conditions. Scale bars, 100 μm (main panels), 50 μm (insets).

Development • Supplementary information

Table S1. Pairwise comparison *p*-values for Fig. 2F and Supplementary Fig. 7 (MBP), as well as for Fig. 2G, and Supplementary Fig. 8 (CC1). Pairwise comparisons between the conditions for MBP signal intensity (*A*) and CC1 signal intensity (*B*) were performed by ANOVA with repeated measures and posthoc LSD. The *p*-values for each nonredundant comparison are shown, and significant differences ($p \leq 0.05$) indicated by an asterisk (*).

**A**	Demyelin-ation regimen	Control + Scrambled shRNA + ONC	Demyelination regimen + Scrambled shRNA + ONC	Control + Anti-Pten shRNA + ONC	Demyelination regimen + Anti-Pten shRNA + ONC
Control	0.00 *	0.02 *	0.01 *	0.00 *	0.00 *
Demyelination regimen		0.06	0.03 *	0.02 *	0.02 *
Control + Scrambled shRNA + ONC			0.12	0.35	0.43
Demyelination regimen + Scrambled shRNA + ONC				0.07	0.69
Control + Anti-Pten shRNA + ONC					0.01 *

**B**	Demyelin-ation regimen	Control + Scrambled shRNA + ONC	Demyelination regimen + Scrambled shRNA + ONC	Control + Anti-Pten shRNA + ONC	Demyelination regimen + Anti-Pten shRNA + ONC
Control	0.96	0.02 *	0.11	0.02 *	0.14
Demyelination regimen		0.01 *	0.12	0.02 *	0.12
Control + Scrambled shRNA + ONC			0.01 *	0.64	0.00 *
Demyelination regimen + Scrambled shRNA + ONC				0.05 *	0.89
Control + Anti-Pten shRNA + ONC					0.02 *

Table S2. Pairwise comparison *p*-values for Figs. 5 and 8 on axon regeneration. Pairwise comparisons between the conditions for pre-treatment with the demyelination regimen (*A*) and post-injury treatment with intravitreal injections (*B*) were performed by ANOVA with repeated measures and posthoc LSD. The *p*-values for each nonredundant comparison are shown, and significant differences ($p \leq 0.05$) indicated by an asterisk (*).

**A**	Control + Anti-Pten shRNA + ONC	Demyelination regimen	Demyelination regimen + Anti-Pten shRNA + ONC
Control	0.00 *	0.02 *	0.00 *
Control + Anti-Pten shRNA + ONC		0.34	0.05 *
Demyelination regimen			0.07

**B**	100 µM cuprizone	1 mM cuprizone	10 mM cuprizone
Saline	0.11	0.05 *	0.24
100 µM cuprizone		0.69	0.58
1 mM cuprizone			0.63

Development • Supplementary information

Table S3. Pairwise comparison *p*-values for Figs. 6C-D on RGC survival. Pairwise comparisons between the conditions for pre-treatment with the demyelination regimen (*A*) and post-injury treatment with intravitreal injections (*B*) were performed by ANOVA with repeated measures and posthoc LSD. The *p*-values for each nonredundant comparison are shown, and significant differences ($p \leq 0.05$) indicated by an asterisk (*).

**A**	Demyelination regimen + Scrambled shRNA + ONC	Control + Anti-Pten shRNA + ONC	Demyelination regimen + Anti-Pten shRNA + ONC
Control + Scrambled shRNA + ONC	0.10	0.38	0.01 *
Demyelination regimen + Scrambled shRNA + ONC		0.37	0.09
Control + Anti-Pten shRNA + ONC			0.02 *

**B**	100 μM cuprizone	1 mM cuprizone	10 mM cuprizone
Saline	0.21	0.24	0.21
100 μM cuprizone		0.93	1.00
1 mM cuprizone			0.93

Development • Supplementary information
