# Supplementary material for: Post-injury born oligodendrocytes incorporate into the glial scar and contribute to the inhibition of axon regeneration
Source: Development. 2023 Apr 27;150(8):dev201311. doi: 10.1242/dev.201311 (PMC10163352; doi:10.1242/dev.201311)
Supplement: Supplementary file 1 [file develop-150-201311_review_history.pdf]

Post-injury born oligodendrocytes incorporate into the glial scar and contribute to the inhibition of axon regeneration

Jian Xing, Agnieszka Lukomska, Bruce A. Rheaume, Juhwan Kim, Muhammad S. Sajid, Ashiti Damania and Ephraim F. Trakhtenberg
DOI: 10.1242/dev.201311

Editor: Steve Wilson

Review timeline

Original submission:	20 September 2022
Editorial decision:	14 November 2022
First revision received:	6 February 2023
Editorial decision:	6 March 2023
Second revision received:	6 March 2023
Accepted:	14 March 2023

Original submission

First decision letter

MS ID#: DEVELOP/2022/201311

MS TITLE: Post-injury born oligodendrocytes integrate into the glial scar and inhibit axon regeneration

AUTHORS: Jian Xing, Bruce A Rheaume, Agnieszka Lukomska, Juhwan Kim, Muhammad S Sajid, Ashiti Damania, and Ephraim F Trakhtenberg

Many apologies for the long time that it took us to obtain reviews on your manuscript. I have now received all the referees' reports on the above manuscript, and have reached a decision. The referees' comments are appended below, or you can access them online: please go to BenchPress and click on the 'Manuscripts with Decisions' queue in the Author Area.

As you will see, the referees express considerable interest in your work, but have some significant criticisms and suggestions toimproveyour manuscript. If you are able to revise the manuscript along the lines suggested, I will be happy receive a revised version of the manuscript. Please note that Development will normally permit only one round of major revision. If it would be helpful, you are welcome to contact us to discuss your revision in greater detail. Please send us a point-by-point response indicating your plans for addressing the referee’s comments, and we will look over this and provide further guidance.

Please attend to all of the reviewers' comments and ensure that you clearly highlight all changes made in the revised manuscript. Please avoid using 'Tracked changes' in Word files as these are lost in PDF conversion. I should be grateful if you would also provide a point-by-point response detailing how you have dealt with the points raised by the reviewers in the 'Response to Reviewers' box. If you do not agree with any of their criticisms or suggestions please explain clearly why this is so.

Reviewer 1

Advance summary and potential significance to field

© 2023. Published by The Company of Biologists under the terms of the Creative Commons Attribution License (https://creativecommons.org/licenses/by/4.0/).

The manuscript by Xing, Lukomska, and Rheaume et al. is addressing the question of why axons that undergo regeneration after CNS injury stall along their way to their target. The hypothesis is that newly generated oligodendrocytes contribute to the inhibition of axon regeneration. They tested this hypothesis by first showing that newly born oligodendrocytes contribute to the glial scar. Somewhat surprisingly, a demyelination treatment along with axon regeneration promoted axon regeneration. The authors conclude that this enhanced regeneration is due to the lack of newborn oligodendrocytes in the glial scar after demyelination treatment. This result is intriguing considering several ongoing clinical trials aim to provide oligodendrocyte progenitors into the injured spinal cord. This study suggests that if done at the wrong time, this stem cell treatment could have a negative impact on any endogenous axon regeneration that may be occurring naturally. The results from this manuscript will be of interest to researchers working on optic nerve regeneration, spinal cord regeneration, traumatic brain injury, and developmental neurobiologists.

Comments for the author

Some general questions when reading the introduction:
Are the inhibitory molecules of primed-to-myelinate oligodendrocytes different than the classic myelin inhibitory molecules CSPG, MAG, NogoA, OMgp, and Semaphorins?

One question that came up is what happens to the existing oligodendrocytes in the optic nerve past the injury site? Do they eventually regress and die without an axon to myelinate? Does the debri from dying oligodendrocytes not inhibit axon regeneration in the region past the glial scar?...These two points could be brought up, but it is up to the authors. I realize this is in the discussion, but was in my head in the introduction

In the introduction, it is argued that the axons can grow past the glial scar but then stall on their way to the target. But, the experiments are focused on NFO's contribution to the glial scar. I suggest restating this argument. Possibly that the presence of NFO's within the glial scar specifically, likely has a significant role in inhibiting axon regeneration past the glial scar.

Results section: In general, the sentences are too long, making it very difficult to interpret. I recommend splitting many of the sentences into at least two, sometimes three to increase readability.

Sentence starting in "Here, we show substantial TUNEL.." has several grammatical errors and is very hard to follow. It should be split into at least two if not three sentences.

-Figure 1 needs to be re-arranged as the images are out of order from the text.

-This sentence "...similar to density in an uninjured optic nerve (Fig. 1A-B)." is referring to TUNEL staining in after ONC, but Fig. 1A-B is of EdU uninjured spinal cord

- Please rephrase this sentence " These observations are consistent with that oligodendrocyte progenitor cells...".

-The injury and AAV scheme is presented in Figure 2A, but Pten experiments are not discussed in the results until Figure 5. This makes it difficult to interpret Figure 2. I suggest either removing Figure 2A, moving it, or duplicated a portion of it later in the manuscript.

-The scRNAseq results are discussed in the results section " Spontaneous clearance of myelin debris from the injury site by 2 weeks after ONC", but this should not be discussed until the section "Changes in oligodendrocyte subpopulations in the glial scar revealed by scRNA-seq". When I read linearly through the manuscript, it is makes it confusing to see the results of this experiment show up before the section it is presented in. Also, the sentence "
Accordingly, using scRNA-seq analysis, we found a 14-fold increase in the immune cell population..." describing this is very long. I suggest splitting this up into three sentences

-Please add a statement that Pdgfra is a marker for OPCs in this sentence
"...population of Pdgfra+ OPCs"

© 2023. Published by The Company of Biologists under the terms of the Creative Commons Attribution License (https://creativecommons.org/licenses/by/4.0/).

-Figure 2F and 2G are not referenced in the text until after Figure 3. Please rearrange figures colinearly with the text

-In the sentence " in the injury site after ONC, Fyn+ NFOs", please include that Fyn is a marker for NFO's

-This sentence is too long to follow: " We hypothesized that the demyelination regimen would diminish...". I recommend splitting.

-Add that it is CTB labelling to figure 5. I realize it says it in the legend but it is helpful to have that information in the figure.

-I suggest adding a star or something to indicate significance of the 1mM dose in Figure 8 "...but only the 1 mM dose was significant"

Discussion-

Several possible future avenues are discussed, but I think it is worth mentioning the possibility of using transgenic reporter lines to label pre ONC and post ONC oligodendrocytes rather than using EdU and IHC as the markers for NFO's. Although the data seem convincing that a significant number of NFOs are formed after ONC some genetic approaches could be considered.

Methods-

Overall, the methods are described clearly and thoroughly.

Reviewer 2

Advance summary and potential significance to field

This manuscript by the Trakhtenberg group (Xing et al) performed scRNAseq in the injured optic nerve (ONC) and characterized the response of the oligodendrocyte lineage after injury; in addition their show the appearance of post-injury born immature newly formed oligodendrocytes (NFO) that seem to integrate into the scar and their role in the regeneration failure after optic nerve crush. Two demyelination regimens with cuprizone are used which reduced the immature and mature oligodendrocyte population after ONC and stimulated some modest optic nerve regeneration (less than 2.5mm). This regeneration was observed when Pten was deleted at the same time (a classic paradigm to promote the endogenous growth propensity of these cells).

The authors propose that post-injury born newly formed oligodendrocytes integrate into the glial scar, where they are susceptible to the demyelination treatments; this reduced their integration in the glial scar and facilitates some modest axonal regeneration.

Comments for the author

In general, this is an interesting paper, a lot of work (in particular the regeneration experiments) and it provides some novel insights into the role of a recently identified immature state of “newly formed oligodendrocytes” (mostly documented by CC1 staining here but also identified by Fyn expression in the scRNA-seq data). These NOFs are found in the lesion where it seems to be inhibitory to regeneration.
The authors showed a convincing albeit modest regeneration effect here. The expression of axonal growth inhibitory molecules by this population is also evident (=described) in the scRNAseq data and their website are great tools for the field. A double staining of these CC1 cells with Fyn in the lesion scar would have made this paper stronger.

Much of the data hinges on the demyelination with cuprizone which is only incompletely understood and affects the entire system with its toxicity. The authors recognize this limitation and perform cuprizone injections into the eye as well – which does not get around the need to characterize the effects of the non-oligodendrocyte lineage. The authors assessed the effects on retinal ganglion

© 2023. Published by The Company of Biologists under the terms of the Creative Commons Attribution License (https://creativecommons.org/licenses/by/4.0/).

cell survival (only few survive in this model anyway) and they provided a superficial account of the inflammatory response.

The latter suggests that the demyelination effect it not likely affecting regeneration via the well established inflammatory effects (that comparison to zymosan is actually provided). Hence, they conclude effects are coming from these immature oligodendrocytes which are reduced after cuprizone treatment. A genetic deletion/interference with oligodendrocyte maturation would have been more satisfying.

What is completely left open is the possible role of the NG2 cells / OPCs and the expression of CSPGs (astrocytes as well) in the context of these experiments. Does the demyelination affect these as well? Could these account for some of the effects on axon sprouting?
Why were the OPC data in Figure 10 with the inhibitory molecules not shown? The server has the expression data for their expression of inhibitory molecules and CSPG4 is notoriously high. Therefore, a discussion of the NG2 cells and their possible role here would improve the paper, ideally with the inclusion of some NG2 immunohistochemistry (with PDGFRa) and other CSPGs. Is there an effect on fibroblast?

We are left with the observation that a “demyelination regimen” would delay the formation of new myelin in the injury site (in the ocular injection of cuprizone paradigm) but also clear the pre-existing myelin and its debris (in the pretreatment paradigm). This is interesting but the multiple mechanisms by which this might occur remain to be discussed at the very least.

Style and writing

It might me by personal bias but the enormously long sentences in the writing style is tedious and could/should be simplified. There are some grammatical errors due to this too. So some language editing is recommended.

Language – word choices:
Cuprizone is not a model for MS and neither is ONC a model for optic neuropathy.
MS is much different and merely shares some aspects of the demyelination and remyelination.
It would be better to just state what it is: Cuprizone or optic nerve crush Similarly, any speculation of using Cuprizone in patients seems highly premature in the light of these modest regeneration data shown here. I would not go there in a basic biology paper.
When the authors talk about “integration” of the NFOs into the glial scar (based on CC1 staining), it seems that the location of the CC1 cells is in the centre of the lesion, in a location distinct from the astocytes; the latter seem to be at the border to the nerve. Hence my hesitation to accept “integration” as the right term? Also there are other immature oligodendrocyte lineage cells staining for CC1.
Calling them post injury born oligodendrocyte lineage cells seems appropriate based on the EdU data.

Typos:
Typo in legend Fig 3 A chow not chaw

Strange language at times: page 8 …… where MBP detection by immunostaining is otherwise borders noise (Fig4). Page 8. Bordering noise?

Page 11: Our data shows. Data is plural

Page 16 Z-stack not stock

<u>Reviewer 3</u>

Advance summary and potential significance to field

© 2023. Published by The Company of Biologists under the terms of the Creative Commons Attribution License (https://creativecommons.org/licenses/by/4.0/).

The manuscript by Xing et al. investigates the reason behind a previously described event where regenerating axons, that respond to regenerative treatments, tend to stop re-growing before reaching their targets.

The authors set out to test the hypothesis that the interaction between the axons (that are stimulated to regenerate) and the newly formed oligodendrocytes (NFOs - which were absent during developmental axon growth) is behind the inefficient axonal regeneration.

Comments for the author

The authors use state-of-the-art animal models and methods to report very interesting findings. However, the manuscript in its present form is written in a very confusing manner. I urge the authors to revise the manuscript and think carefully about its organization to be able to get a fair review of a lot of interesting data that they produced. In its present format, the data is lost in the middle of a such a disorganized manuscript.

Just a few examples:
-the rational between the choice of what is a main figure and what is a supplementary one should be re-evaluated. For instance, the description of the gene browser should go to supplementary data as it cuts the flow of the experimental rational;
-in the results section the authors are jumping figures. For instance, some of the results of figure 3 are presented before the formal description of how these results were obtained and the same with figure 2 that is referred early on in the manuscript but only two sections below is the methodology explained;
-the title of some of the result sections should be revised. For example, the title “Demyelination regimen remodels the glial scar after traumatic optic nerve injury” does not seem to correspond to what is described in there.

On a more scientific note, I would draw the attention to the following points:
Fig. 3 - using a single cell RNA-seq the authors describe a pseudotimeline trajectory from OPCs into NFOs and then into the primed-to-myelinate sSOs, and finally into the fully mature oligodendrocytes in the context of ONC injury model.

However, it is not clear why was Pdgrfa and Fyn used to define OPCs and NFO populations, respectively. It would be nice to show these same markers in the injured nerve fiber to corroborate the data obtained in figure 1.

Page 6 – do not understand the need or relevance to provide an extensive description of what happens in corpus callosum tracts in the main text. This is a detour that distracts from the main massage of this manuscript.

Fig.10 – what is MO1, MO2 and MO3? how were these results obtained? Are these coming from the scRNA seq? Are these qPRC data?

First revision

Author response to reviewers' comments

POINT-BY-POINT RESPONSE TO CRITIQUES

Please find below our responses to the reviewers’ comments. The reviewers raised excellent points that helped to improve the manuscript. The revised manuscript addresses all of the reviewers’ critiques, as detailed below.

REVIEWER 1

© 2023. Published by The Company of Biologists under the terms of the Creative Commons Attribution License (https://creativecommons.org/licenses/by/4.0/).

Advance Summary and Potential Significance to Field: The manuscript by Xing, Lukomska, and Rheaume et al. is addressing the question of why axons that undergo regeneration after CNS injury stall along their way to their target. The hypothesis is that newly generated oligodendrocytes contribute to the inhibition of axon regeneration. They tested this hypothesis by first showing that newly born oligodendrocytes contribute to the glial scar. Somewhat surprisingly, a demyelination treatment along with axon regeneration promoted axon regeneration. The authors conclude that this enhanced regeneration is due to the lack of newborn oligodendrocytes in the glial scar after demyelination treatment. This result is intriguing considering several ongoing clinical trials aim to provide oligodendrocyte progenitors into the injured spinal cord. This study suggests that if done at the wrong time, this stem cell treatment could have a negative impact on any endogenous axon regeneration that may be occurring naturally. The results from this manuscript will be of interest to researchers working on optic nerve regeneration, spinal cord regeneration, traumatic brain injury, and developmental neurobiologists.

Reviewer 1 Comments for the Author:

Some general questions when reading the introduction:

Are the inhibitory molecules of primed-to-myelinate oligodendrocytes different than the classic myelin inhibitory molecules CSPG, MAG, NogoA, OMgp, and Semaphorins?

--- In the revised Fig. 10, we performed comparative analysis on the expression of classic myelin inhibitory molecules in the primed-to-myelinate oligodendrocytes and mature oligodendrocytes (MOs). We showed classic myelin inhibitors, NogoA (a.k.a. Rtn4), OMgp, MAG, and Sema5A, and added a discussion on other inhibitory Semaphorins, which were not expressed in the primed-to-myelinate oligodendrocytes. We did not include CSPG, because oligodendrocyte lineage cells are known to express CSPG4 (a.k.a. NG2) and CSPG5, which are not inhibitory to axon growth, whereas the inhibitory CSPGs are secreted by reactive astrocytes. However, it was established in the multiple sclerosis field that cuprizone is not toxic to astrocytes (also see our response to R2).

One question that came up is what happens to the existing oligodendrocytes in the optic nerve past the injury site? Do they eventually regress and die without an axon to myelinate? Does the debri from dying oligodendrocytes not inhibit axon regeneration in the region past the glial scar? These two points could be brought up, but it is up to the authors. I realize this is in the discussion, but was in my head in the introduction.

--- We added the following phrase in the Introduction section, where we briefly address the surviving mature oligodendrocytes located beyond the injury site: “they may provide the inhibitory myelin debris if they die at a later time”. We also further elaborated on this point in the Discussion section.

In the introduction, it is argued that the axons can grow past the glial scar, but then stall on their way to the target. But, the experiments are focused on NFO's contribution to the glial scar. I suggest restating this argument. Possibly that the presence of NFO's within the glial scar specifically, likely has a significant role in inhibiting axon regeneration past the glial scar.

--- We edited to clarify this point, by adding the following phrases in the Introduction “the presence of newly born oligodendrocytes within the glial scar could inhibit axon regeneration past the glial scar”, and “optic nerve axons experimentally-induced to regenerate after injury can interact with newly born oligodendrocytes in the glial scar and become myelinated even while they are still growing, which eventually could contribute to stalling axon growth even past the glial scar”. We also specified in the Abstract about the NFOs being in the glial scar.

Results section: In general, the sentences are too long, making it very difficult to interpret. I recommend splitting many of the sentences into at least two, sometimes three to increase readability.

--- We edited to make sentences shorter.

© 2023. Published by The Company of Biologists under the terms of the Creative Commons Attribution License (https://creativecommons.org/licenses/by/4.0/).

Sentence starting in "Here, we show substantial TUNEL." has several grammatical errors and is very hard to follow. It should be split into at least two if not three sentences.

--- We edited into shorter sentences.

-Figure 1 needs to be re-arranged as the images are out of order from the text.

--- We re-arranged so that more panels are now in proper order, but panel E is still out of order. To fit this panel in order, the panels would need to be made smaller, otherwise it would not fit the page. We believe that it would be better to preserve visibility in the current size panels, but if R1 still believes that we should prioritize order, then we will reduce panels and rearrange further.

-This sentence "...similar to density in an uninjured optic nerve (Fig. 1A-B)." is referring to TUNEL staining after ONC, but Fig. 1A-B is of EdU uninjured optic nerve.

--- We meant to say that Olig2-positive cells that are distal from the injury site (in the figure where we showed TUNEL/Olig2 co-labeling) are found there at a similar density as in an uninjured optic nerve (where we did EdU/Olig2 co-labeling), which is consistent with us not finding TUNEL signal distal from the injury site but only in the injury site. We were pointing to Olig2-poisitve cells, which are comparable between these two figures. We edited that paragraph to make this clearer.

- Please rephrase this sentence "These observations are consistent with that oligodendrocyte progenitor cells...".

--- We edited to rephrase.

-The injury and AAV scheme is presented in Figure 2A, but Pten experiments are not discussed in the results until Figure 5. This makes it difficult to interpret Figure 2. I suggest either removing Figure 2A, moving it, or duplicated a portion of it later in the manuscript.

--- We duplicated portions of Figure 2A later in the manuscript, where it was relevant (new panels A in Figs. 4, 5, 8). We also reduced Figure 2A legend to what was relevant for Fig. 2, as other parts moved to later figures.

-The scRNAseq results are discussed in the results section "Spontaneous clearance of myelin debris from the injury site by 2 weeks after ONC", but this should not be discussed until the section "Changes in oligodendrocyte subpopulations in the glial scar revealed by scRNA-seq". When I read linearly through the manuscript, it is makes it confusing to see the results of this experiment show up before the section it is presented in.

--- We agree, but the discussion on the immune cells in the injury site was relevant to section "Spontaneous clearance of myelin debris from the injury site by 2 weeks after ONC", that is why we included just that part of the larger scRNA-seq discussion there. If we include the full discussion of the scRNA-seq there, it would disrupt the flow of that section. Thus, in order to resolve this issue, where we mention this in the "Spontaneous clearance of myelin debris from the injury site by 2 weeks after ONC" section, we added in parenthesis “(see detailed discussion of the scRNA-seq analysis in the next section)”. We can edit further if needed.

Also, the sentence "Accordingly, using scRNA-seq analysis, we found a 14-fold increase in the immune cell population..." describing this is very long. I suggest splitting this up into three sentences.

--- We edited into shorter sentences.

-Please add a statement that Pdgfra is a marker for OPCs in this sentence "...population of Pdgfra+ OPCs".

--- We added this in the revised manuscript.

© 2023. Published by The Company of Biologists under the terms of the Creative Commons Attribution License (https://creativecommons.org/licenses/by/4.0/).

-Figs. 2F and 2G are not referenced in the text until after Fig. 3. Please rearrange figures colinearly with the text.

--- We edited this, and referenced Figure 2F and 2G in a relevant sentence before Figure 3.

-In the sentence "in the injury site after ONC, Fyn+ NFOs", please include that Fyn is a marker for NFOs.

--- We added this in the revised manuscript.

-This sentence is too long to follow: "We hypothesized that the demyelination regimen would diminish...". I recommend splitting.

--- We edited into shorter sentences.

-Add that it is CTB labelling to figure 5. I realize it says it in the legend, but it is helpful to have that information in the figure.

--- We added CTB label into the Fig. 5.

-I suggest adding a star or something to indicate significance of the 1mM dose in Figure 8 "...but only the 1 mM dose was significant".

--- We added *p*-values table and a star in Fig. 8.

Discussion-
Several possible future avenues are discussed, but I think it is worth mentioning the possibility of using transgenic reporter lines to label pre ONC and post ONC oligodendrocytes rather than using EdU and IHC as the markers for NFO's. Although the data seem convincing that a significant number of NFOs are formed after ONC, some genetic approaches could be considered.

--- We added suggestion of this point in the Discussion section in the revised manuscript.

Methods-
Overall, the methods are described clearly and thoroughly.

REVIEWER 2

Advance Summary and Potential Significance to Field: This manuscript by the Trakhtenberg group (Xing et al) performed scRNAseq in the injured optic nerve (ONC) and characterized the response of the oligodendrocyte lineage after injury; in addition they show the appearance of post-injury born immature newly formed oligodendrocytes (NFO) that seem to integrate into the scar and their role in the regeneration failure after optic nerve crush. Two demyelination regimens with cuprizone are used which reduced the immature and mature oligodendrocyte population after ONC and stimulated some modest optic nerve regeneration (less than 2.5mm). This regeneration was observed when Pten was deleted at the same time (a classic paradigm to promote the endogenous growth propensity of these cells). The authors propose that post-injury born newly formed oligodendrocytes integrate into the glial scar, where they are susceptible to the demyelination treatments; this reduced their integration in the glial scar and facilitates some modest axonal regeneration.

Reviewer 2 Comments for the Author:

In general, this is an interesting paper, a lot of work (in particular the regeneration experiments) and it provides some novel insights into the role of a recently identified immature state of "newly formed oligodendrocytes" (mostly documented by CC1 staining here but also identified by Fyn expression in the scRNA-seq data). These NFOs are found in the lesion where it seems to be inhibitory to regeneration. The authors showed a convincing albeit modest regeneration effect here. The expression of axonal growth inhibitory molecules by this population is also evident (=described) in the scRNAseq data and their website are great tools for the field. A double staining of

© 2023. Published by The Company of Biologists under the terms of the Creative Commons Attribution License (https://creativecommons.org/licenses/by/4.0/).

these CC1 cells with Fyn in the lesion scar would have made this paper stronger.

--- We immunostained in the injured optic nerve for CC1 and Fyn, and added data in new Supplementary Fig. 2.

Much of the data hinges on the demyelination with cuprizone which is only incompletely understood and affects the entire system with its toxicity. The authors recognize this limitation and perform cuprizone injections into the eye as well – which does not get around the need to characterize the effects on the non-oligodendrocyte lineage. The authors assessed the effects on retinal ganglion cell survival (only few survive in this model anyway) and they provided a superficial account of the inflammatory response.

The latter suggests that the demyelination effect it not likely affecting regeneration via the well-established inflammatory effects (that comparison to zymosan is actually provided). Hence, they conclude effects are coming from these immature oligodendrocytes which are reduced after cuprizone treatment. A genetic deletion/interference with oligodendrocyte maturation would have been more satisfying.

--- We added in the Discussion section a future direction for genetic deletion/interference with oligodendrocyte maturation. Our findings using cuprizone are nevertheless also useful for other reasons. For example, cuprizone diet is widely used to injure/kill oligodendrocytes in the multiple sclerosis field. Thus, our findings using cuprizone will have broad implications, including for the multiple sclerosis field. Also, not many labs that study axon regeneration could afford conditional-deletion mice so they often use the more affordable alternative of zymosan, whereas our finding that intralocular injection of cuprizone promotes axon regeneration provides an affordable way to stimulate axon regeneration for studying underlying molecular mechanisms without eliciting inflammation (like zymosan does).

What is completely left open is the possible role of the NG2 cells / OPCs and the expression of CSPGs (astrocytes as well) in the context of these experiments. Does the demyelination affect these as well? Could these account for some of the effects on axon sprouting? Why were the OPC data in Figure 10 with the inhibitory molecules not shown? The server has the expression data for their expression of inhibitory molecules and CSPG4 is notoriously high. Therefore, a discussion of the NG2 cells and their possible role here would improve the paper, ideally with the inclusion of some NG2 immunohistochemistry (with PDGFRa) and other CSPGs.

--- We added new Supplementary Figure 9, with the injured optic nerves after cuprizone or without cuprizone immunostained for Pdgrfa (to label OPCs) and astrocytic CSPG (that inhibits axon growth), which shows that cuprizone did not apparently decrease Pdgrfa-labeled OPCs or astrocytic CSPG labeling in the injury site, which is consistent with prior reports that cuprizone is toxic selectively to oligodendrocytes.

As R2 noted, expression of CSPG4 is notoriously high in the OPCs based on our scRNAseq data, which is consistent with that CSPG4 encodes NG2 marker of the OPCs, as the R2 also noted. However, there is a long history in the field on whether NG2/OPCs have a positive or negative effect, or no effect, on axon regeneration, with different groups reporting different results. We did not feel that our paper would be well-suited for focusing on that, as our focus was on the NFOs. That is why, while we provided new Supplementary Figure 9, as well as the OPC data as a resource for the community through the scRNAseq gene browser we developed, we preferred not to get into that question deeper and did not include the OPCs into our analysis in Fig. 10. The labs that specialize in that specific question can now use our scRNAseq data to assist with their NG2/OPC studies. However, we added the NG2/OPC point R2 raised in the Discussion section, where we clarify that our scRNAseq data provides resource for further investigation of this question. If R2 still feels that we should include OPCs into the Fig. 10, we will of course do it too.

Although based on prior literature CSPG4/NG2 and CSPG5 from the OPCs do not inhibit CNS axon regeneration *in vivo*, other CSPGs secreted by reactive astrocytes after injury were established as inhibitors of axon regeneration (e.g., astrocytic CSPG for which we immunostained in the new Supplementary Figure 9). Because in the multiple sclerosis field, cuprizone was established not to be toxic to astrocytes (and if anything, indirectly activate them, which would hinder axon

© 2023. Published by The Company of Biologists under the terms of the Creative Commons Attribution License (https://creativecommons.org/licenses/by/4.0/).

regeneration), we did not think that cuprizone promoted axon regeneration through acting on astrocytes, and that is why we did not pursue this question deeper. However, we edited the Discussion section to clarify this point, and we also generated new data shown in new Supplementary Fig. 9, where we immunostained the injured optic nerves for Pdgrfa and CSPG after cuprizone or without cuprizone, and we did not find apparent reduction in either Pdgrfa-labeled OPCs or in astrocytic CSPG labeling, which is consistent with prior reports of that cuprizone is toxic selectively to oligodendrocytes.

Is there an effect on fibroblast?

--- Based on our scRNAseq, fibroblasts population was quite small relative to other cell types in the optic nerve, and we did not find meaningful changes in fibroblasts population after injury, as we showed in Fig. 3C-E. Also, in the multiple sclerosis field, cuprizone was established to be toxic only to oligodendrocytes but not to fibroblasts or other cell types in the CNS. That is why we did not analyze it further with markers after cuprizone treatment.

We are left with the observation that a “demyelination regimen” would delay the formation of new myelin in the injury site (in the ocular injection of cuprizone paradigm) but also clear the pre-existing myelin and its debris (in the pretreatment paradigm). This is interesting but the multiple mechanisms by which this might occur remain to be discussed at the very least.

--- In terms of clearance of the pre-existing myelin and its debris (in the pretreatment paradigm), we find that the pre-existing myelin in uninjured optic nerve is reduced only by 10% in the pretreatment paradigm. It also appears that macrophages spontaneously fill the injury site (like we showed by scRNAseq in Fig. 3C-E, and by Iba1 immunostaining in the injured optic nerve in Supplementary Fig. 3) and clear the myelin debris from the injury site by 2 weeks after optic nerve injury, regardless of pretreatment with cuprizone diet.

We clarified in the Discission section that, because cuprizone is established in the multiple sclerosis field to injure/kill oligodendrocytes, and we find that newly formed oligodendrocytes after injury in the optic nerve are especially susceptible to cuprizone, that is why new myelin formation is delayed after cuprizone intraocular injection. We also clarified in the Discission section that, injury and death of NFOs due to cuprizone injection is the main mechanism, and that after cuprizone exits the ocular system then NFOs formation can resume.

Style and writing.

It might me by personal bias but the enormously long sentences in the writing style is tedious and could/should be simplified. There are some grammatical errors due to this too. So some language editing is recommended.

--- We edited to make sentences shorter and corrected grammatical errors. See specific examples of corrections we made in our responses below, and also in our responses to R1 and R3, who pointed-out specific examples where long sentences needed to be shortened and grammatical errors needed to be corrected.

Language – word choices:

Cuprizone is not a model for MS and neither is ONC a model for optic neuropathy. MS is much different and merely shares some aspects of the demyelination and remyelination. It would be better to just state what it is: Cuprizone or optic nerve crush.

--- We edited not to refer to cuprizone as a model for MS, and just say that it shares some aspects of demyelination with MS. We also edited not to use phrase “ONC is a model for traumatic optic neuropathy”, but clarified that ONC is often used to study traumatic optic neuropathy and later just say optic nerve crush or injury.

Similarly, any speculation of using Cuprizone in patients seems highly premature in the light of these modest regeneration data shown here. I would not go there in a basic biology paper.

© 2023. Published by The Company of Biologists under the terms of the Creative Commons Attribution License (https://creativecommons.org/licenses/by/4.0/).

--- We edited to clarify in the Discussion that we are not suggesting to use cuprizone in patients.

When the authors talk about "integration" of the NFOs into the glial scar (based on CC1 staining), it seems that the location of the CC1 cells is in the centre of the lesion, in a location distinct from the astrocytes; the latter seem to be at the border to the nerve. Hence my hesitation to accept "integration" as the right term?

--- We edited to replace "integration" with words like "present in" and "incorporate into".

Also there are other immature oligodendrocyte lineage cells staining for CC1. Calling them post injury born oligodendrocyte lineage cells seems appropriate based on the EdU data.

--- We edited by adding the phrase "oligodendrocyte lineage cells", in most places where we mention post injury born immature oligodendrocytes.

Typos:

--- We edited the manuscript to correct the typos listed below.

Typo in legend Fig 2 A chow, not chaw.

Strange language at times: page 8 …… where MBP detection by immunostaining is otherwise borders noise (Fig4). Page 8. Bordering noise?

Page 11: Our data shows. Data is plural.

Page 16 Z-stack, not stock.

REVIEWER 3

Advance Summary and Potential Significance to Field: The manuscript by Xing et al. investigates the reason behind a previously described event where regenerating axons, that respond to regenerative treatments, tend to stop re-growing before reaching their targets.

The authors set out to test the hypothesis that the interaction between the axons (that are stimulated to regenerate) and the newly formed oligodendrocytes (NFOs - which were absent during developmental axon growth) is behind the inefficient axonal regeneration.

Reviewer 3 Comments for the Author:

The authors use state-of-the-art animal models and methods to report very interesting findings. However, the manuscript in its present form is written in a very confusing manner. I urge the authors to revise the manuscript and think carefully about its organization to be able to get a fair review of a lot of interesting data that they produced. In its present format, the data is lost in the middle of a such a disorganized manuscript.

--- We edited the manuscript based on the feedback from each of the 3 reviewers, all of whom raised this issue and made specific suggestions on how to improve organization of the manuscript.

Just a few examples:

-the rational between the choice of what is a main figure and what is a supplementary one should be re- evaluated. For instance, the description of the gene browser should go to supplementary data as it cuts the flow of the experimental rational;

--- We removed the section which describes the gene browser, and integrated its description into the relevant portions of the Discussion and Methods sections.

© 2023. Published by The Company of Biologists under the terms of the Creative Commons Attribution License (https://creativecommons.org/licenses/by/4.0/).

-in the results section the authors are jumping figures. For instance, some of the results of figure 3 are presented before the formal description of how these results were obtained, and the same with figure 2 that is referred early on in the manuscript but only two sections below in the methodology explained;

--- Regarding Figure 3 being referred earlier in the manuscript than where its results are fully explained later in the manuscript (which is an issue noted by R1 too), we apologize for the confusion. We did that because the discussion on the scRNA-seq data on immune cells in the injury site was relevant to an earlier section ("Spontaneous clearance of myelin debris from the injury site by 2 weeks after ONC"), but the full results of the Figure 3 were reported later for a comprehensive scRNA-seq analysis of the oligodendrocytes lineage cells. If we would include the full analysis of the scRNA-seq data earlier (where we just wanted to bring-up inferences regarding the immune cells), this would have disrupted the flow of that section, since the bulk of the scRNA-seq results are about the oligodendrocytes and only little is about the immune cells. Thus, in order to resolve this issue, where we mention scRNA-seq data from Figure 3 earlier in the manuscript (under the section "Spontaneous clearance of myelin debris from the injury site by 2 weeks after ONC"), we added in parenthesis “(see detailed discussion of the scRNA-seq analysis in the next section)”. We can edit further if needed.

Regarding the issue of some of the results of Figure 2 being presented before the description of methodology two sections later, we apologize for confusing the readers by showing a figure that includes demyelination regimen before we actually introduced this method in the main text. The parts of Figure 2 referenced in text before we explained the methodology of demyelination regimen, were actually for the data generated from the control groups that did not undergo demyelination regimen, that is why we did not explain the demyelination regimen methodology earlier, but we now see how this is confusing, because demyelination regimen label is throughout the Figure 2 panels. To resolve this issue, where Figure 2 is first referenced in the last paragraph of the first section, we edited and added in parenthesis along with a clarification: “(also see control conditions after ONC in Fig. 2B-G; treatment conditions are described in later sections)”. Similarly, two other times where Figure 2 is referenced in the following sections prior to its full explanation (i.e., methodology of the demyelination regimen and all parts of the Figure 2), we also edited and added in parenthesis along with a clarification: “(see control conditions after ONC in Fig. 2B-G; treatment conditions are described in later sections)”. We can edit further if needed.

-the title of some of the result sections should be revised. For example, the title “Demyelination regimen remodels the glial scar after traumatic optic nerve injury” does not seem to correspond to what is described in there.

--- We edited the title of that section to “Effect of demyelination regimen on traumatically injured optic nerve”.

On a more scientific note, I would draw the attention to the following points:

Fig. 3 - using a single cell RNA-seq the authors describe a pseudotimeline trajectory from OPCs into NFOs and then into the primed-to-myelinate sSOs, and finally into the fully mature oligodendrocytes in the context of ONC injury model. However, it is not clear why was Pdgrfa and Fyn used to define OPCs and NFO populations, respectively. It would be nice to show these same markers in the injured nerve fiber to corroborate the data obtained in figure 1.

--- We selected Pdgrfa and Fyn to define OPCs and NFO populations, because Ben Barres used these markers for the same purpose in the Journal of Neuroscience 2014 paper (which we cited), titled “An RNA-Sequencing Transcriptome and Splicing Database of Glia, Neurons, and Vascular Cells of the Cerebral Cortex”. We immunostained the injured optic nerves for Pdgrfa and Fyn, and added this new data in new Supplementary Figures 2 and 9.

Page 6 – do not understand the need or relevance to provide an extensive description of what happens in corpus callosum tracts in the main text. This is a detour that distracts from the main massage of this manuscript.

© 2023. Published by The Company of Biologists under the terms of the Creative Commons Attribution License (https://creativecommons.org/licenses/by/4.0/).

--- We just wanted to show that cuprizone diet worked well in our hands, like in other papers that used cuprizone, which mostly looked at corpus callosum. Because loss of myelin in corpus callosum in our study is similar to that in those other studies, the results we obtained for the optic nerve are reliable. We edited to clarify.

Fig.10 – what is MO1, MO2 and MO3? how were these results obtained? Are these coming from the scRNA seq? Are these qPRC data?

--- MO1, MO2 and MO3 are three subtypes of mature oligodendrocytes (MOs), which we obtained by cluster analysis of the optic nerve scRNAseq data we generated. Although not qPCR, prior studies (which we cited) also found several subtypes of MOs in other brain regions by scRNAseq cluster analysis. We edited to clarify.

Second decision letter

MS ID#: DEVELOP/2022/201311

MS TITLE: Post-injury born oligodendrocytes incorporate into the glial scar and contribute to the inhibition of axon regeneration

AUTHORS: Jian Xing, Bruce A Rheaume, Agnieszka Lukomska, Juhwan Kim, Muhammad S Sajid, Ashiti Damania, and Ephraim F Trakhtenberg

You will be pleased to hear that the reviewers are happy with your revisions and there are just a few minor issues to respond to before we proceed to publication. The referees' comments are appended below, or you can access them online: please go to BenchPress and click on the 'Manuscripts with Decisions' queue in the Author Area.

Reviewer 1

Advance summary and potential significance to field

The authors have responded in a satisfactory manner and addressed all concerns raised by reviewers.

Comments for the author

None

Reviewer 2

Advance summary and potential significance to field

This reviewer goes along with the revisions and changes in the resubmission.

Comments for the author

NO further changes suggested.

Reviewer 3

Advance summary and potential significance to field

© 2023. Published by The Company of Biologists under the terms of the Creative Commons Attribution License (https://creativecommons.org/licenses/by/4.0/).

The manuscript by Xing et al. investigates the reason behind a previously described event where regenerating axons, that respond to regenerative treatments, tend to stop re-growing before reaching their targets.

The authors set out to test the hypothesis that the interaction between the axons (that are stimulated to regenerate) and the newly formed oligodendrocytes (NFOs - which were absent during developmental axon growth) is behind the inefficient axonal regeneration. They generated convincing evidence for the inhibitory effect on axonal re-growth exerted by new oligodendrocytes at the injury site.

Comments for the author

This is an improved version of the manuscript, much more coherent and organized.
The authors addressed my criticisms, however there are a few minor things:
1.
Figure 1F
The name of the markers used in each individual panel needs to be included to facilitate the interpretation of the figure.

2.
Figure 10
The legend should refer that these data were obtained from scRNA-seq.
And although the authors refer to extra information in Supplementary File 1, what is MO1 MO2 and MO3 is still missing. How were these defined?

3.
The authors should further improve the spelling and grammar check throughout the manuscript.
There are still several typos across the text, like:
positve, labled, psudotimeline, just to name a few and same sentences that need re-writing like this one where the adverb is missing:
“By 3 days after ONC, we find only a few oligodendrocytes are visualized by CC1…”

Second revision

<u>Author response to reviewers' comments</u>

POINT-BY-POINT RESPONSE TO CRITIQUES

Please find below our response to the Reviewer 3 comments (Reviewers 1 and 2 did not have any further comments). The reviewer noted issues, which we corrected and thereby improved the manuscript. The revised manuscript addresses all of the issues, as detailed below.

REVIEWER 3

Reviewer 3 Advance Summary and Potential Significance to Field:

The manuscript by Xing et al. investigates the reason behind a previously described event where regenerating axons, that respond to regenerative treatments, tend to stop re-growing before reaching their targets. The authors set out to test the hypothesis that the interaction between the axons (that are stimulated to regenerate) and the newly formed oligodendrocytes (NFOs - which were absent during developmental axon growth) is behind the inefficient axonal regeneration. They generated convincing evidence for the inhibitory effect on axonal re- growth exerted by new oligodendrocytes at the injury site.

© 2023. Published by The Company of Biologists under the terms of the Creative Commons Attribution License (https://creativecommons.org/licenses/by/4.0/).

Reviewer 3 Comments for the Author:

This is an improved version of the manuscript, much more coherent and organized. The authors addressed my criticisms, however there are a few minor things:

1. Figure 1F. The name of the markers used in each individual panel needs to be included to facilitate the interpretation of the figure.

--- We apologize for overlooking this. The names of the markers are now added in the revised Figure 1F individual panels.

2. Figure 10. The legend should refer that these data were obtained from scRNA-seq. And although the authors refer to extra information in Supplementary File 1, what is MO1 MO2 and MO3 is still missing. How were these defined?

--- The original revised manuscript exceeded allowed word count, and that is why we had to move a part of the Figure 10 legend to the Supplementary File 1. However, we have now received permission from the Editor to move extra information for Figure 10 legend from the Supplementary File 1 back to the main text Figure 10 legend. We further edited to clarify in the figure legend that these data were obtained from scRNA-seq, and explained how MO1, MO2, and MO3 were defined.

3. The authors should further improve the spelling and grammar check throughout the manuscript. There are still several typos across the text, like: positve, labled, psudotimeline, just to name a few and same sentences that need re-writing like this one where the adverb is missing: “By 3 days after ONC, we find only a few oligodendrocytes are visualized by CC1…”

--- We apologize for the typos. We edited the manuscript to correct typos, and also corrected the sentences that needed re-writing.

<u>Third decision letter</u>

MS ID#: DEVELOP/2022/201311

MS TITLE: Post-injury born oligodendrocytes incorporate into the glial scar and contribute to the inhibition of axon regeneration

AUTHORS: Jian Xing, Bruce A Rheaume, Agnieszka Lukomska, Juhwan Kim, Muhammad S Sajid, Ashiti Damania, and Ephraim F Trakhtenberg
ARTICLE TYPE: Research Article

I am happy to tell you that your manuscript has been accepted for publication in Development, pending our standard ethics checks.

© 2023. Published by The Company of Biologists under the terms of the Creative Commons Attribution License (https://creativecommons.org/licenses/by/4.0/).
